# Supplementary material for: Chemical warfare between leafcutter ant symbionts and a co-evolved pathogen
Source: Nat Commun. 2018 Jun 7;9:2208. doi: 10.1038/s41467-018-04520-1 (PMC5992151; doi:10.1038/s41467-018-04520-1)
Supplement: Supplementary file 1 — Supplementary Information [file 41467_2018_4520_MOESM1_ESM.pdf]

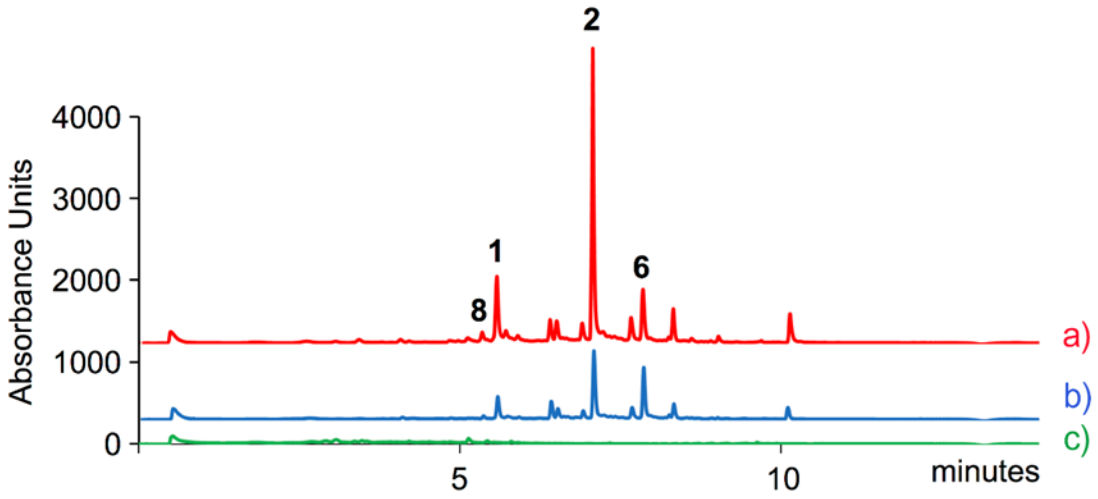

Supplementary Figure 1 | *Escovopsis* pathogenesis involves upregulation of small molecule virulence factors. UPLC profiles (λ_{abs} 254 nm) of ethyl acetate extracts of a) *L. gongylophorus* infected with *E. weberi* strain A; b) *E. weberi* strain A axenic culture; and c) *L. gongylophorus* axenic culture. Compounds are: **1** = melinacidin IV; **2** = shearinine D; **6** = shearinine A; and **8** = emodin. This experiment was carried out independently in the Hutchings/Wilkinson lab (Norwich), but the results are similar to those from the experiment carried out in Jena (Fig. 2, main text). Agar plates (9 cm diameter) containing 25 ml R5 agar [1] were each inoculated with 100 µl spore stock solution of *Escovopsis weberi* strain A. For the co-cultivation experiments, freshly ground *L. gongylophorus* fungus garden (1 g) was distributed over an R5 agar plate that was freshly inoculated with *Escovopsis weberi* as described above. Equivalent *L. gongylophorus* monoculture plates were prepared in the same way (without *E. weberi*). The plates were incubated for 7 days at room temperature in the dark. The agar was then chopped into small pieces and extracted with ethyl acetate (5 ml). The material was filtered, the filtrate evaporated under reduced pressure and dissolved in methanol (1.5 ml) for subsequent UPLC (UV & MS) analysis. Analytical UPLC was carried out on an Accela UPLC system (Thermo Scientific) using a Kinetex® 1.7 µm C18 100 Å, 100 x 2.1 mm column (Phenomenex). The mobile phase was a mixture of solvent A (0.1% formic acid in water) and solvent B (methanol) with a gradient as follows: solvent A/B initial condition 90/10, hold at 90/10 for 1 min, linear gradient up to 0/100 within 9.00 min, hold for 2.00 min, returned to 90/10 within 0.5 min, hold at 90/10 for 1.5 min; flow rate was 0.6 ml min^{-1}. Mean area of **1** for the co-culture (n=2; 1839548 au^2) and for the axenic culture (n=2 ; 849645 au^2); mean area of **2** for the co-culture (n=2 ; 6431606 au^2) and for the axenic culture (n=2 ; 2221102 au^2).

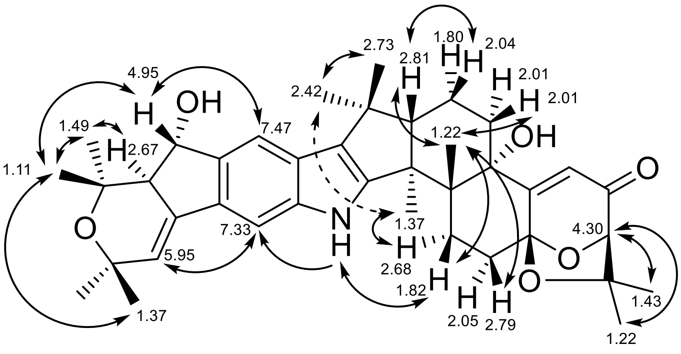

Supplementary Figure 2 | NOESY NMR data for shearinine D (2) confirming its relative configuration.

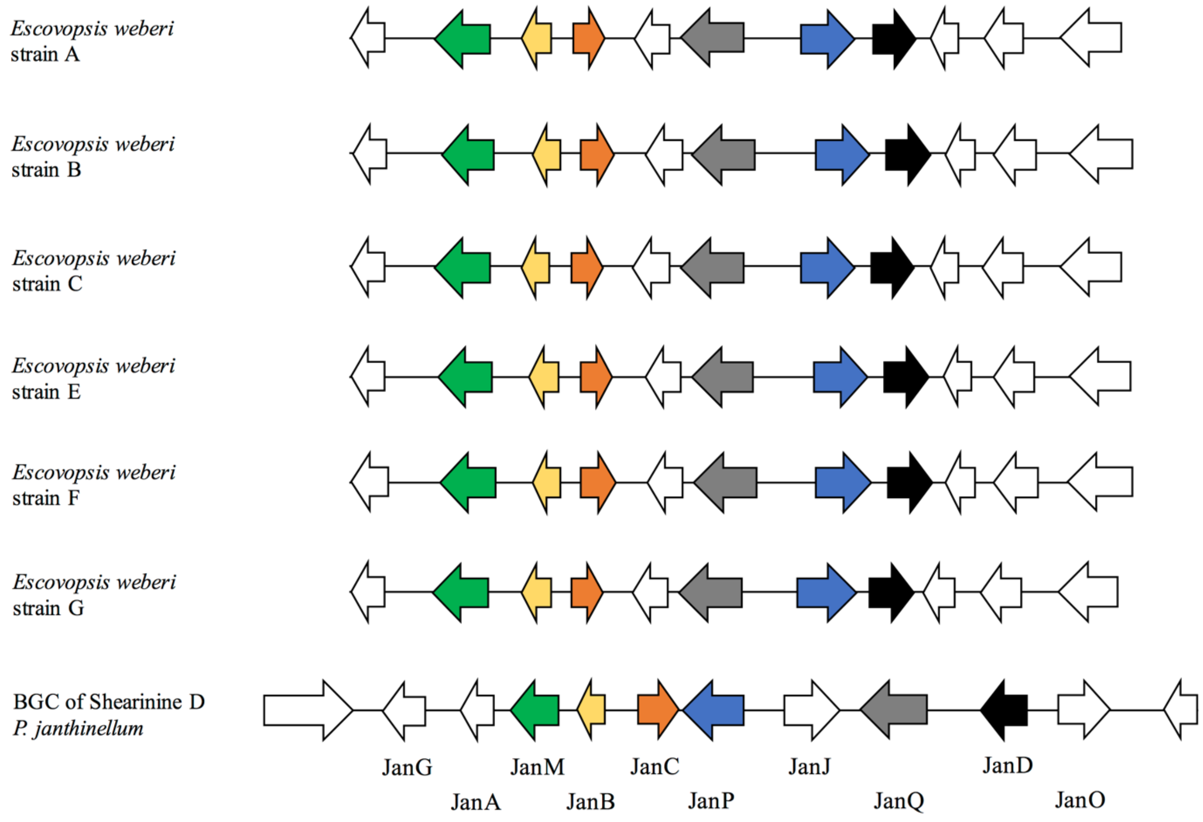

Supplementary Figure 3 | Analysis of the terpene-indole alkaloid biosynthetic gene clusters (BGCs) present in the genomes of sequenced *Escovopsis* strains (Supplementary Table 1). The *Escovopsis* BGCs share similarity to the BGC for the production of shearinines in *P. janthinellum* [2]. While the genes *janB-D*, *janM*, *janP* and *janQ* constitute part of the BGC in these strains, homologues for *janG* (a geranylgeranyl pyrophosphate synthase) and *janO* (an FAD dependent monooxygenase) occur at a distinct location in the genome (KOS21612.1 and KOS22754.1, respectively in strain G). No homologues could be identified for *janA* and *janJ*. No such BGC is present in strain D. The annotated BGC from strain G was given the accession number TPA: BK010418.

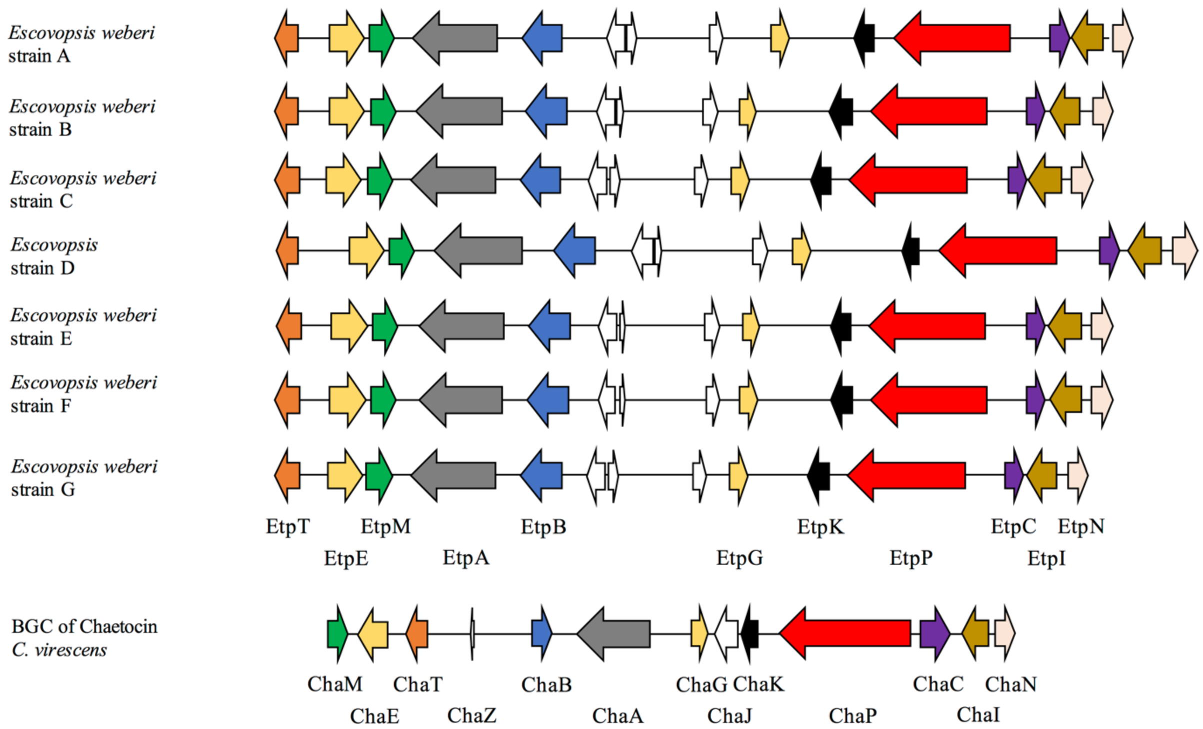

Supplementary Figure 4 | Analysis of the epipolythiodiketopiperazine (ETP) BGCs present in the genomes of sequenced *Escovopsis* strains. ETP BGCs are present in all the sequenced *Escovopsis* strains. The *Escovopsis* BGCs share similarity to the BGC for production of the ETP chaetocin in *C. virescens* [3]. Homologues for the putative regulator *chaZ* and dipeptidase *chaJ* could not be identified. The annotated BGC from strain G was given the accession number TPA: BK010420.

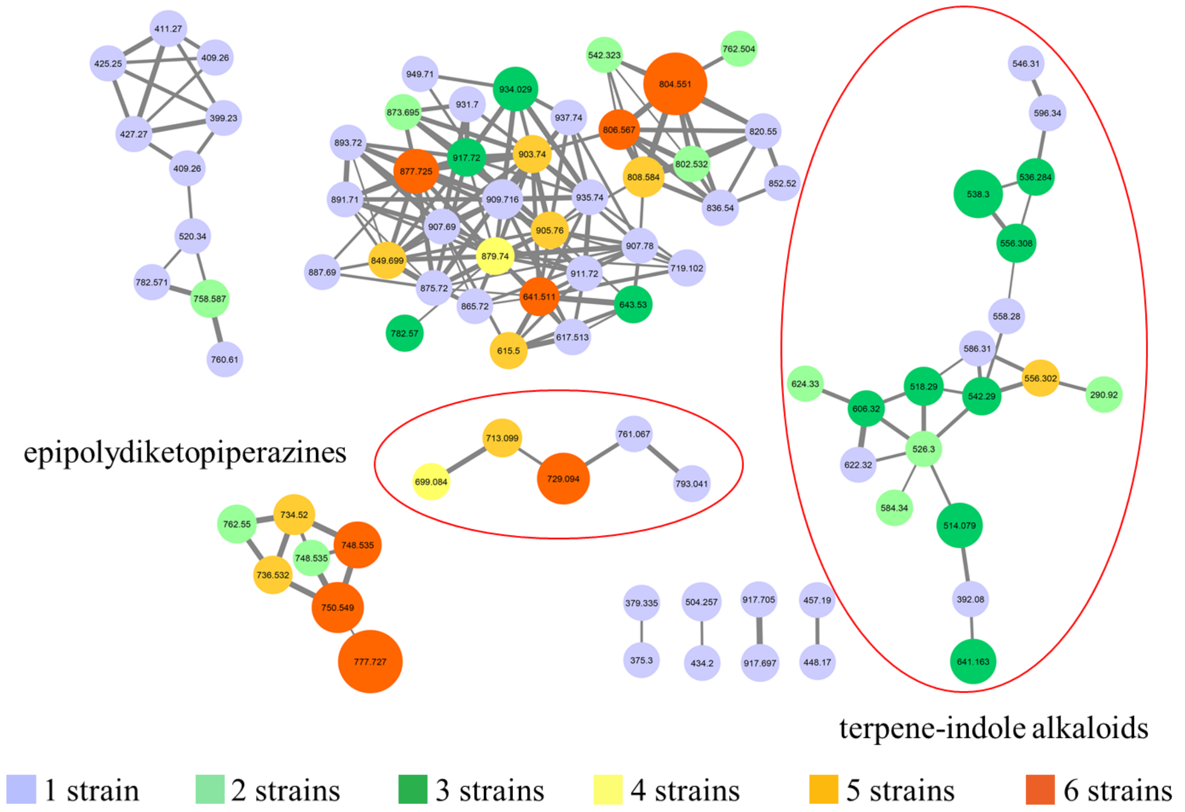

Supplementary Figure 5 | Global metabolomics analysis of *Escovopsis* strains. Molecular network of metabolites of *Escovopsis* strains A-F. The size of each node represents the number of spectra present for a specific feature. The color of each node represents the number of strains that share the respective metabolite. The ETP and terpene-indole alkaloid networks are highlighted; the remaining networks relate to unidentified natural product families produced by *Escovopsis* strains.

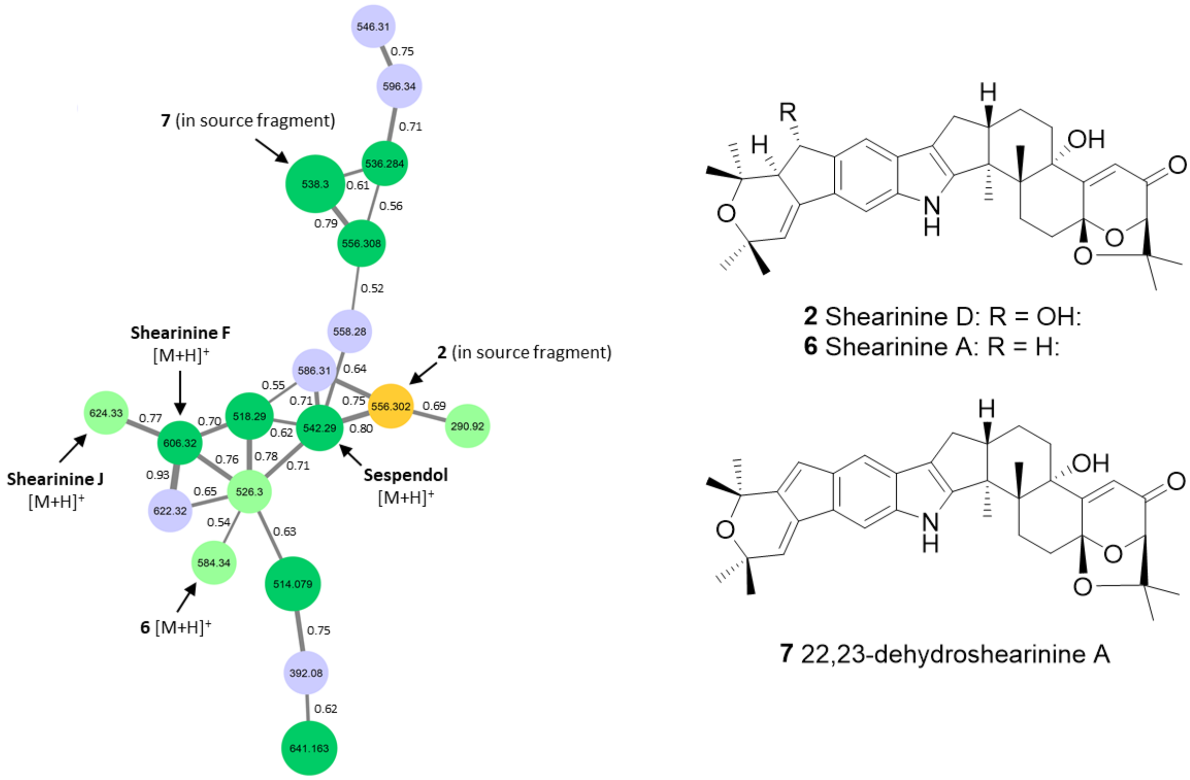

Supplementary Figure 6 | Molecular network and chemical structures of terpene-indole alkaloids being produced by *E. weberi* strains A-C, E and F.

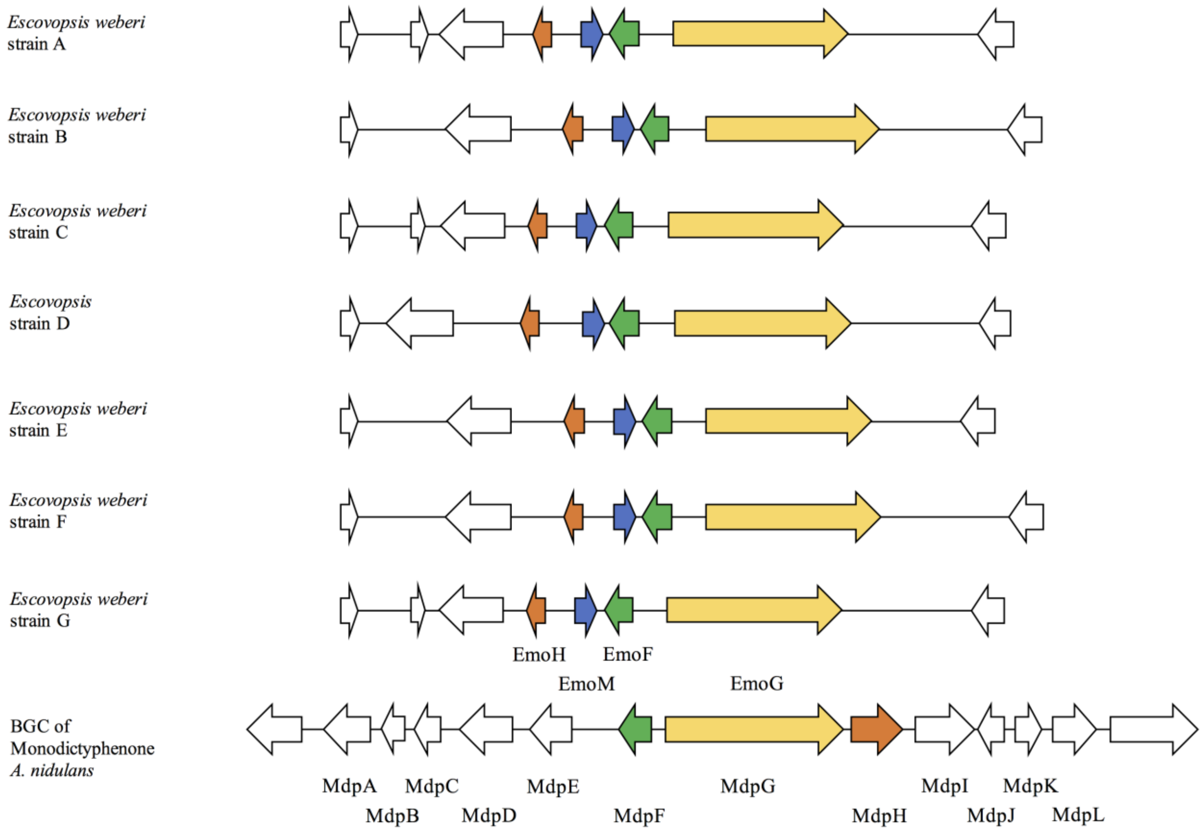

Supplementary Figure 7 | Analysis of the proposed emodin BGCs present in the genomes of sequenced *Escovopsis* strains. Emodin BGCs are present in all of the sequenced *Escovopsis* strains. The *Escovopsis* BGCs share similarity to the BGC for production of monodictyphenone in *Aspergillus nidulans* [4]. Emodin is an intermediate in the monodictyphenone pathway and as such the emodin BGC is stripped down to contain only the genes necessary for production of emodin. A PKS can form the tricyclic precursor to emodin followed by a beta-lactamase-like enzyme that performs an analogous function to a thioesterase and hydrolyses altrochrysone carboxylic acid from the acyl carrier protein domain. Decarboxylation (by EmoH) and dehydration (by EmoM) complete emodin production. The annotated cluster from strain G was given the accession number TPA: BK010419.

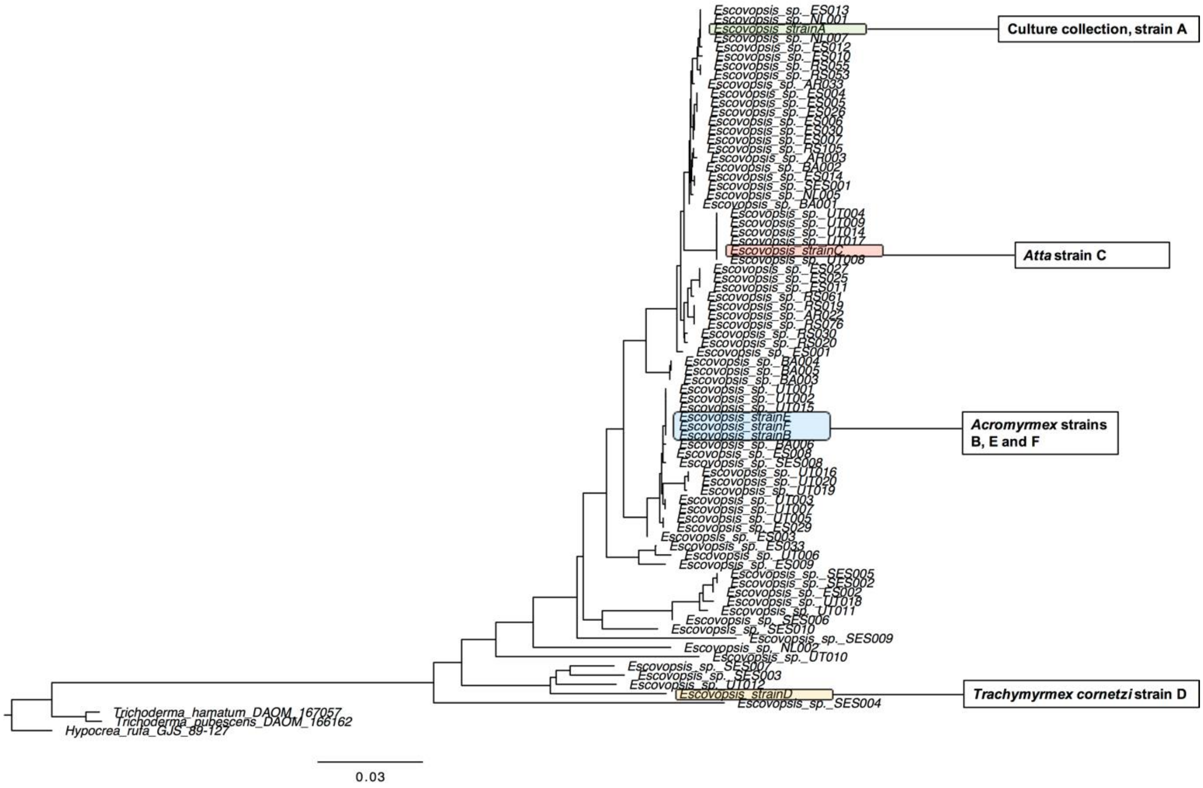

Supplementary Figure 8 | Phylogenetic analysis of *Escovopsis* strains. Clustal Omega (1.2.4) [5] was used to generate alignments of *tef1* gene sequences from *Escovopsis* strains A-F and those recovered from GenBank (accession codes: KM817113 to KM817173, KF240730, KF240731, EU082795 to EU082803, AF534585, AF534620, AF534624 [6,7,8]). Similarly alignments were made with internal transcriber spacer (ITS) gene sequences from *Escovopsis* strains A-F and those recovered from Genbank (accession codes: KM817043 to KM817112, X93980, EU280124, NR_077179 [7,9,10]). Alignments were concatenated using WINCLADA (ver 1.61)[11]. A phylogenetical tree was constructed using FigTree v1.4.3 2016: http://tree.bio.ed.ac.uk/software/figtree/. *Hypocrea rufa* GJS 89-127 was selected as the root. The sequences for strains A-F and their sources are highlighted. The scale bar represents 3% sequence divergence. The ITS sequence for *Escovopsis* strain D was amplified with primers ITS1 and ITS4, sequenced and deposited with Genbank accession: MG897412.

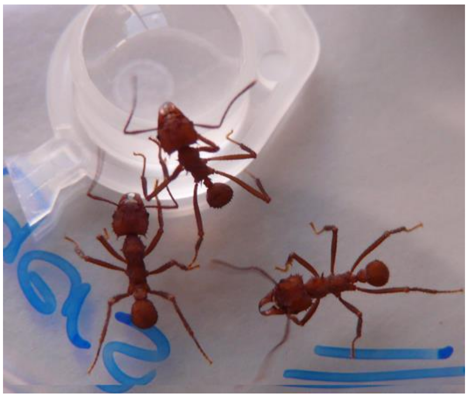

Supplementary Figure 9 | *Acromyrmex echinatior* worker ants feeding from an Eppendorf cap filled with a 5% glucose solution supplemented with compound 2.

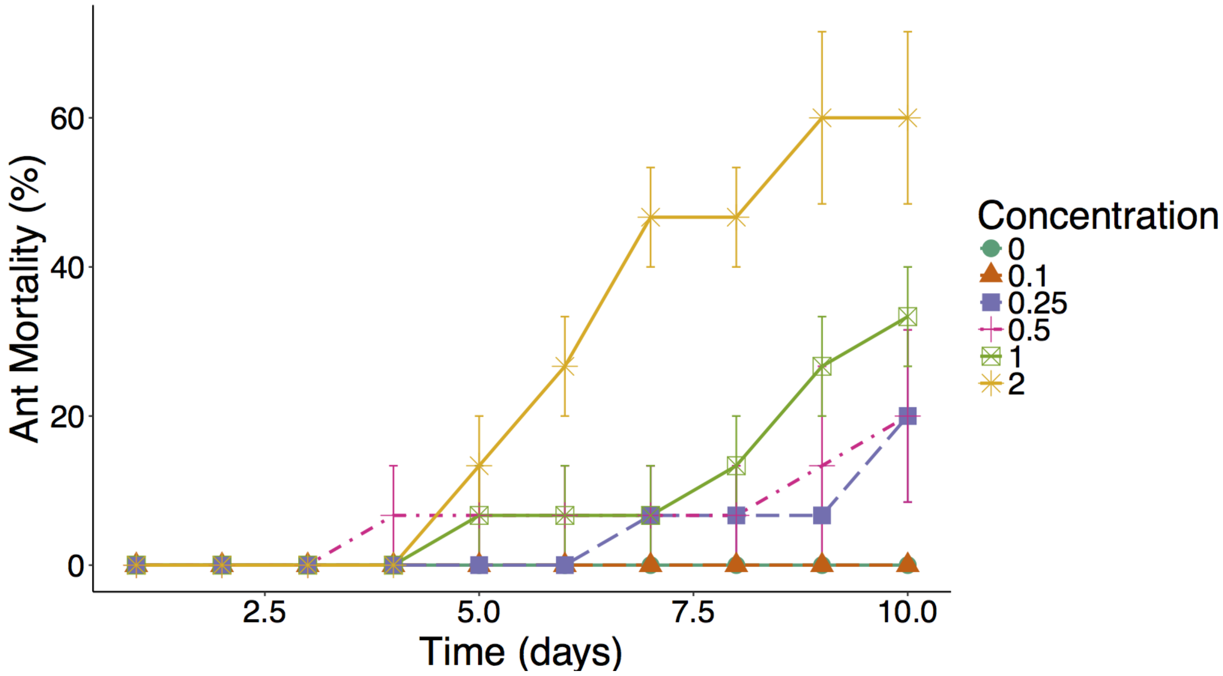

Supplementary Figure 10 | Average cumulative percentage mortality of worker ants in experimental groups receiving different concentrations (mM) of compound 2 over 10 days. Values represent the average percentage mortality in groups of ants across three replicate runs of the experiment (error bars ± SE; *n* = 5 ants per treatment level, in each run of the experiment).

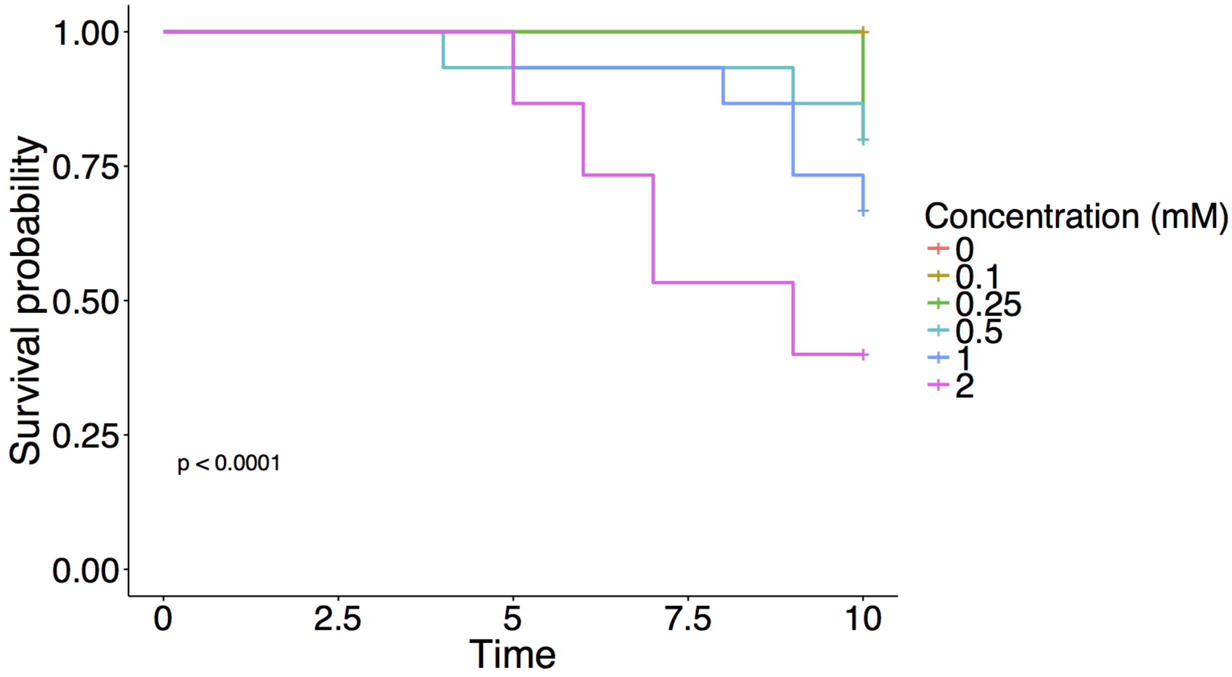

Supplementary Figure 11 | Kaplan Meier survival curve indicating the probability of ant survival over time and at various concentrations (mM) of compound 2. A Cox's mixed effects model indicated that higher concentrations significantly reduced survival probability over time (*P* = <0.001).

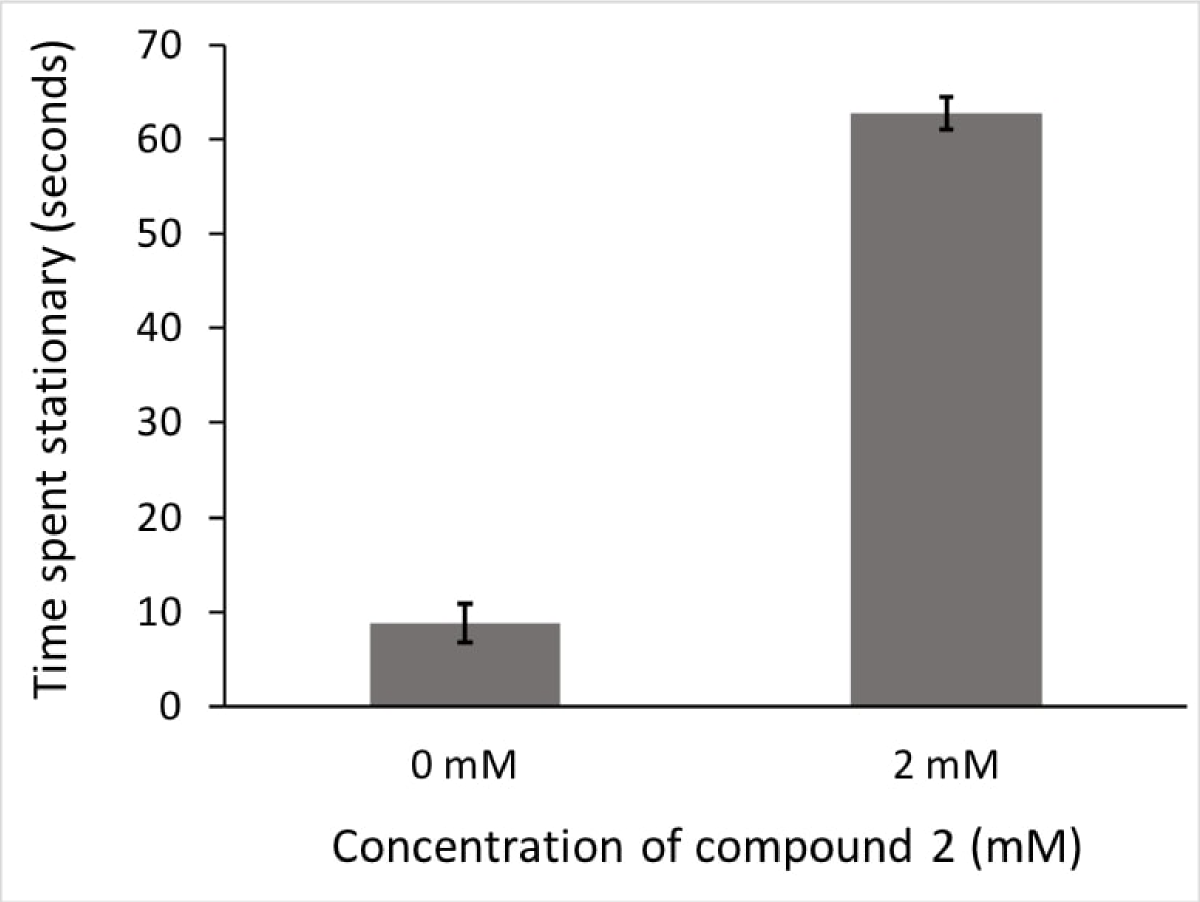

Supplementary Figure 12 | A comparison of the mobility of ants receiving different concentrations of compound 2. The average number of seconds (± SE) spent stationary (out of 65 seconds of film) was calculated from time lapse videos. N = 5 ants in each treatment group. There was a significant difference between the two treatments ($t_{(7.73)}$ = 19.74, P = <0.001, Welch's t-test).

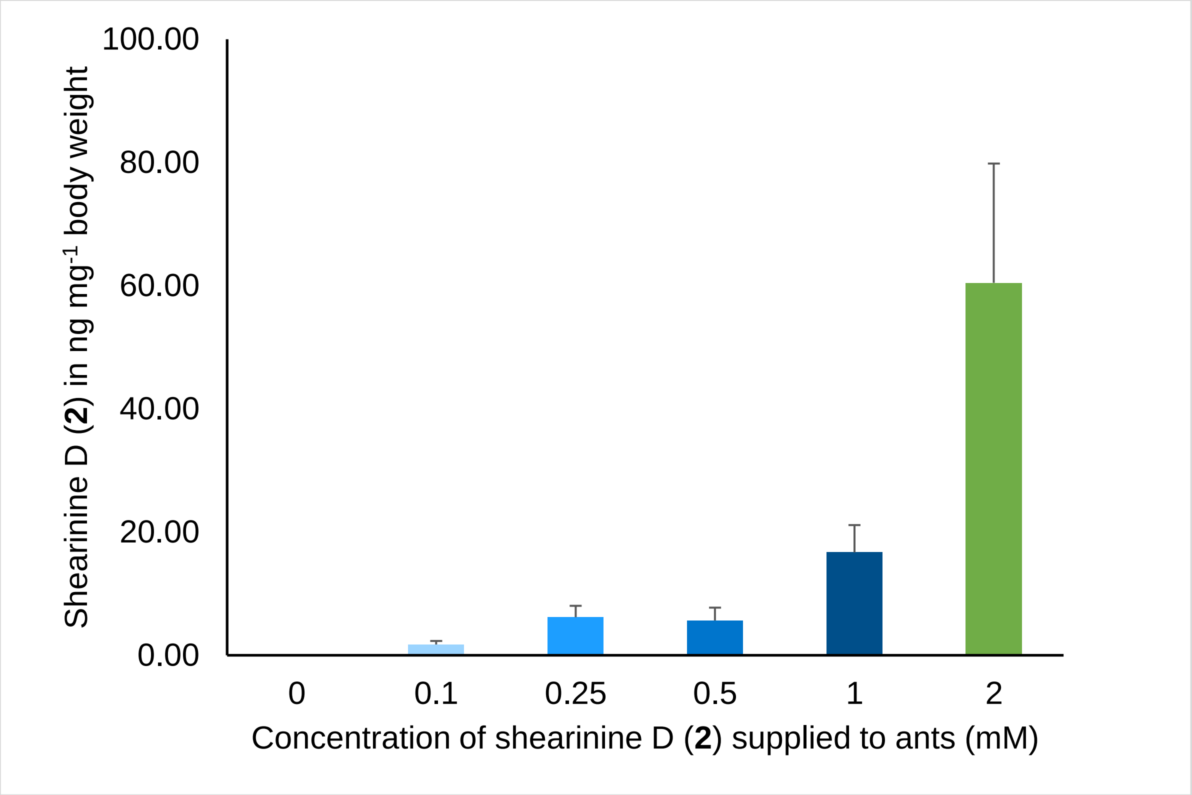

Supplementary Figure 13 | Concentrations of compound 2 (± SE) detected in ants treated with a 5% glucose solution containing various quantities of compounds 2.

**Strain**	**Source**	**Location / Date**	**No. contigs / N50 (Mbp)**	**Genome size (Mbp)**	**GenBank accession code**	**Reference**
A	Isolated from an unknown attine species in Colombia	Unknown	13 / 3.9	27.38	NIGB00000000	Strain CBS 810.71Westerdijk Fungal Biodiversity Institute
B	*Acromyrmex echinatior* colony Ae733	Gamboa, Panama / 2015	25 / 2.26	29.98	NQYR00000000	This study
C	*Atta colombica* colony	Gamboa, Panama / 2015	13 / 4.28	27.14	NQYS00000000	This study
D	*Trachomyrmex cornetzi* colony 177605	Gamboa, Panama / 2015	19 / 6.16	38.32	NIGD00000000	This study
E	*Acromyrmex echinatior* colony Ae720	Gamboa, Panama / 2015	234 / 0.79	31.56	NQYQ00000000	This study
F	*Acromyrmex echinatior* colony Ae724	Gamboa, Panama / 2015	17 / 3.26	29.91	NIGC00000000	This study
G	*Atta cephalotes* colony	Gamboa, Panama. / unknown.	29 / 2.58	27.20	LGSR00000000	Reference 12

Supplementary Table 1 | *Escovopsis* strains used in this study.

***Escovopsis* strain**	Strain A	Strain B	Strain C	Strain E	Strain F	Strain G
**Contig**	SC08	SC03	SC06	SC020	SC02	LGSR01000019.1
**Gene name (predicted function)**	**Strain A position**	**Strain B position**	**Strain C position**	**Strain E position**	**Strain F position**	**Strain G position**<br>**Gene Identifier**
ORF (unknown)	1358116 to 1357058	3174683 to 3173618	109708 to 110768	258204 to 259269	2917808 to 2916743	931768 to 930709<br>ESCO_005796
JanM (FAD-dependent mono-oxygenase)	1361548 to 1359746	3178189 to 3176477	106272 to 108077	254697 to 256410	2921314 to 2919602	935204 to 933399<br>ESCO_005797
JanB (integral membrane protein)	1363497 to 1362565	3180319 to 3179384	104334 to 105256	252565 to 253499	2923444 to 2922509	937138 to 936220<br>ESCO_005798
JanC (prenyl transferase)	1364204 to 1365194	3180994 to 3181997	103624 to 102634	251890 to 250887	2924119 to 2925122	937848 to 938838<br>ESCO_005799
ORF (unknown)	1367362 to 1366223	3184270 to 3183147	100461 to 101600	248614 to 249737	2927392 to 2926269	941008 to 939869<br>ESCO_005800
JanQ (cytochrome P450 mono-oxygenase)	1369762 to 1367784	3186658 to 3184666	98063 to 100041	246226 to 248218	2929779 to 2927787	943406 to 941428<br>ESCO_005801
JanP (cytochrome P450 mono-oxygenase)	1371625 to 1373361	3188604 to 3190329	96220 to 94484	244280 to 242555	2931725 to 2933450	945249 to 946985<br>ESCO_005802
JanD (aromatic prenyl transferase)	1373947 to 1375341	3190925 to 3192343	93898 to 92504	241955 to 240537	2934050 to 2935468	947571 to 948965<br>ESCO_005803
ORF (unknown)	1376706 to 1375837	3193704 to 3192831	91137 to 92007	239176 to 240049	2936829 to 2935956	950332 to 949462<br>ESCO_005804

ORF (unknown)	1378830 to 1377531	3195749 to 3194407	89011 to 90313	237134 to 238473	2938871 to 2937532	952457 to 951156<br>ESCO_005805
ORF (unknown)	1381992 to 1379995	3198854 to 3196857	85848 to 87845	234031 to 236028	2941976 to 2939979	955620 to 953623<br>ESCO_005806

Supplementary Table 2 | Position of genes and predicted function for the terpene-indole alkaloid BGCs in the genomes of sequenced *Escovopsis* strains. The annotated BGC from strain G was given the accession number TPA: BK010418.

***Escovopsis* strain**	Strain G	*P. Janthinellum*<br>Jan Cluster	E value	Identities
**Contig**	LGSR01000019.1	KF280651		
**Gene name (predicted function)**	**Strain G position**	**Gene Name**		
	**Gene Identifier**	**Gene Identifier**		
JanM (FAD-dependent mono-oxygenase)	935204 to 933399	JanM	1e-105	159/390 (41%)
	ESCO_005797	AGZ20480		
JanB (integral membrane protein)	937138 to 936220	JanB	3e-90	127/234 (54%)
	ESCO_005798	AGZ20474		
JanC (prenyl transferase)	937848 to 938838	JanC	1e-98	139/280 (50%)
	ESCO_005799	AGZ20475		
JanQ (cytochrome P450 mono-oxygenase)	943406 to 941428	JanQ	6e-119	185/510 (36%)
	ESCO_005801	AGZ20487		
JanP (cytochrome P450 mono-oxygenase)	945249 to 946985	JanP	2e-131	196/474 (41%)
	ESCO_005802	AGZ20476		
JanD (aromatic prenyl transferase)	947571 to 948965	JanD	1e-34	112/418 (27%)
	ESCO_005803	AGZ20478		
**Contig**	LGSR01000008.1	KF280651		
JanG	230627 to 231592	JanG	2e-119	169/310 (55%)
	ESCO_005024	AGZ20472		
**Contig**	LGSR01000002.1	KF280651		
JanO	653147 to 653800	JanO	5e-158	219/444 (49%)
	ESCO_003538	AGZ20488		

Supplementary Table 3 | BLASTP [13] analysis of the proteins encoded by the terpene-indole alkaloid BGC from *P. janthinellum* [2] and the reference genome of *Escovopsis* strain G. Similarity was assessed according to E value and shared amino acid identities.

Escovopsis **strain**	Strain G	Strain A	E value	Identities	Strain B	E value	Identities	Strain C	E value	Identities	Strain E	E value	Identities	Strain F	E value	Identities
**Contig**	LGSR01000019.1	SC08			SC03			SC06			SC020			SC02		
**Gene name (predicted function)**	**Strain G position**<br>**Gene Identifier**	**Strain A position**			**Strain B position**			**Strain C position**			**Strain E position**			**Strain F position**		
ORF (unknown)	931768 to 930709<br>ESCO_005796	1358116 to 1357058	6e-143, 2e-73	191/201 (95%), 106/111 (95%)	3174683 to 3173618	2e-137, 4e-71	182/196 (93%), 100/111 (90%)	109708 to 110768	0.0	295/322 (92%)	258204 to 259269	4e-138, 4e-71	183/196 (93%), 100/111 (90%)	2917808 to 2916743	2e-137, 4e-71	182/196 (93%), 100/111 (90%)
JanM (FAD-dependent mono-oxygenase)	935204 to 933399<br>ESCO_005797	1361548 to 1359746	0.0, 0.0, 0.0	183/214 (86%), 125/132 (95%), 79/81 (98%)	3178189 to 3176477	0.0	335/476 (70%)	106272 to 108077	0.0, 0.0, 0.0	186/214 (87%), 125/132 (95%), 81/81 (100%)	254697 to 256410	0.0, 0.0	266/360 (74%), 72/85 (85%)	2921314 to 2919602	0.0	335/476 (70%)
JanB (integral membrane protein)	937138 to 936220<br>ESCO_005798	1363497 to 1362565	7e-121, 1e-46	172/174 (99%), 70/71 (99%)	3180319 to 3179384	3e-118, 7e-46	167/174 (96%), 68/71 (96%)	104334 to 105256	5e-127, 5e-47	174/174 (100%), 71/71 (100%)	252565 to 253499	3e-118, 1e-44	167/174 (96%), 68/71 (96%)	2923444 to 2922509	3e-118, 7e-46	167/174 (96%), 68/71 (96%)
JanC (prenyl transferase)	937848 to 938838<br>ESCO_005799	1364204 to 1365194	5e-127, 1e-71	171/171 (100%), 104/105 (99%)	3180994 to 3181997	1e-156, 1e-156	189/200 (95%), 97/105 (92%)	103624 to 102634	5e-127, 1e-71	171/171 (100%), 105/107 (98%)	251890 to 250887	1e-155, 1e-155	189/200 (95%), 96/105 (91%)	2924119 to 2925122	1e-156, 1e-156	189/200 (95%), 97/105 (92%)
ORF (unknown)	941008 to 939869<br>ESCO_005800	1367362 to 1366223	8e-125, 7e-66	204/241 (85%), 110/113 (97%)	3184270 to 3183147	1e-102, 9e-67	168/197 (85%), 100/113 (88%)	100461 to 101600	6e-131, 2e-68	212/241 (88%), 113/113 (100%)	248614 to 249737	3e-101, 9e-67	167/197 (85%), 100/113 (88%)	2927392 to 2926269	8e-107, 9e-67	169/197 (86%), 100/113 (88%)
JanQ (cytochrome P450 mono-oxygenase)	943406 to 941428<br>ESCO_005801	1369762 to 1367784	0.0, 0.0, 0.0, 0.0, 0.0, 0.0	141/142 (99%), 140/144 (97%), 76/78 (97%), 77/99 (78%), 58/59 (98%), 36/36 (100%)	3186658 to 3184666	0.0, 6e-121, 6e-121, 6e-121	257/323 (80%), 136/188 (72%), 64/89 (72%), 33/36 (92%)	98063 to 100041	0.0, 0.0, 0.0, 0.0, 0.0, 0.0	141/142 (99%), 143/144 (99%), 76/78 (97%), 77/99 (78%), 59/60 (98%), 36/36 (100%)	246226 to 248218	0.0, 6e-121, 6e-121, 6e-121	257/323 (80%), 136/188 (72%), 64/89 (72%), 33/36 (92%)	2929779 to 2927787	0.0, 6e-121, 6e-121, 6e-121	257/323 (80%), 136/188 (72%), 64/89 (72%), 33/36 (92%)
JanP (cytochrome P450 mono-oxygenase)	945249 to 946985<br>ESCO_005802	1371625 to 1373361	0.0, 0.0, 0.0, 0.0, 0.0	223/223 (100%), 71/71 (100%), 86/93 (92%), 65/65 (100%),	3188604 to 3190329	0.0, 0.0, 0.0, 9e-38, 2e-27	213/230 (93%), 77/101 (76%), 84/93 (90%), 64/65 (98%),	96220 to 94484	0.0, 0.0, 0.0, 0.0, 0.0	223/223 (100%), 71/71 (100%), 86/93 (92%), 65/65 (100%),	244280 to 242555	0.0, 0.0, 0.0, 9e-38, 2e-27	213/230 (93%), 74/86 (86%), 84/93 (90%), 64/65 (98%),	2931725 to 2933450	0.0, 0.0, 0.0, 9e-38, 2e-27	213/230 (93%), 77/101 (76%), 84/93 (90%), 64/65 (98%),

				51/51 (100%)			48/52 (92%)			51/51 (100%)			48/52 (92%)			48/52 (92%)
JanD (aromatic prenyl transferase)	947571 to 948965<br>ESCO_005803	1373947 to 1375341	0.0, 0.0, 0.0	224/226 (99%), 147/150 (98%), 37/38 (97%)	3190925 to 3192343	2e-141, 2e-89, 1e-21	214/240 (89%), 139/150 (93%), 40/55 (73%)	93898 to 92504	0.0, 0.0, 0.0	225/226 (99%), 149/150 (99%), 38/38 (100%)	241955 to 240537	2e-141, 2e-89, 1e-21	214/240 (89%), 139/150 (93%), 40/55 (73%)	2934050 to 2935468	0.0, 0.0, 1e-21	212/230 (92%), 139/150 (93%), 40/55 (73%)
ORF (unknown)	950332 to 949462<br>ESCO_005804	1376706 to 1375837	7e-130, 7e-130, 7e-130	137/137 (100%), 72/74 (97%), 25/25 (100%)	3193704 to 3192831	8e-117, 8e-117, 8e-117	131/137 (96%), 69/74 (93%), 25/25 (100%)	91137 to 92007	1e-129, 1e-129, 1e-129	137/137 (100%), 74/74 (100%), 25/25 (100%)	239176 to 240049	8e-117, 8e-117, 8e-117	131/137 (96%), 69/74 (93%), 25/25 (100%)	2936829 to 2935956	8e-117, 8e-117, 8e-117	131/137 (96%), 69/74 (93%), 25/25 (100%)
ORF (unknown)	952457 to 951156<br>ESCO_005805	1378830 to 1377531	0.0, 0.97	323/326 (99%), 6/6 (100%)	3195749 to 3194407	0.0, 1.5	310/326 (95%), 6/6 (100%)	89011 to 90313	0.0, 0.97	326/326 (100%), 6/6 (100%)	237134 to 238473	0.0, 1.5	310/326 (95%), 6/6 (100%)	2938871 to 2937532	0.0, 1.5	310/326 (95%), 6/6 (100%)
ORF (unknown)	955620 to 953623<br>ESCO_005806	1381992 to 1379995	0.0	663/665 (99%)	3198854 to 3196857	0.0	630/665 (95%)	85848 to 87845	0.0	665/665 (100%)	234031 to 236028	0.0	630/665 (95%)	2941976 to 2939979	0.0	630/665 (95%)

Supplementary Table 4 | BLASTX [13] analysis of the proteins encoded by the terpene-indole alkaloid BGC from the reference genome of *Escovopsis* strain G and the nucleotide sequences of the terpene-indole alkaloid BGC from *Escovopsis* strains A, B, C, E & F. Similarity was assessed according to E value and shared amino acid identities.

Escovopsis strain	**Strain A**	**Strain B**	**Strain C**	**Strain D**	**Strain E**	**Strain F**	**Strain G**
**Contig**	SC05	SC07	SC05	SC05	SC024	SC10	LGSR01000002.1
**Gene name (predicted function)**	**Strain A**	**Strain B**	**Strain C**	**Strain D**	**Strain E**	**Strain F**	**Strain G**<br>**Gene Identifier**
EtpT (thioredoxin reductase)	324397 to 325642	2133685 to 2134931	2252891 to 2251641	3202227 to 3203420	268585 to 269831	1574269 to 1575515	2232345 to 2231095<br>ESCO_003933
EtpE (cytochrome P450 mono-oxygenase)	322606 to 320855	2131923 to 2130141	2254504 to 2256257	3199415 to 3197632	266791 to 265009	1572507 to 1570725	2233958 to 2235711<br>ESCO_003934
EtpM (methyl transferase)	320487 to 319147	2129695 to 2128413	2256625 to 2257915	3197206 to 3195983	264563 to 263276	1570279 to 1568992	2236079 to 2237369<br>ESCO_003935
EtpA (ABC transporter)	313586 to 318100	2122708 to 2127241	2263511 to 2258997	3190085 to 3194823	257540 to 262104	1563285 to 1567820	2242965 to 2238451<br>ESCO_003936
EtpB (cytochrome P450 mono-oxygenase)	310114 to 312202	2119099 to 2121302	2267129 to 2264955	3186108 to 3188282	254012 to 256130	1559626 to 1561876	2246484 to 2244404<br>ESCO_003937
ORF (unknown)	306645 to 307583	2116530 to 2117474	2269582 to 2268662	3183054 to 3184079	251449 to 252387	1557063 to 1558001	2248934 to 2248014<br>ESCO_003938
ORF (unknown)	306421 to 306002	2116300 to 2116082	2269806 to 2270264	3182743 to 3182519	251219 to 251001	1556833 to 1556615	2249158 to 2249615<br>ESCO_003939
ORF	301951	2111637	2274390	3177538	246518	1552178	2253731 to

(unknown)	to 301202	to 2110942	to 2275115	to 3176795	to 245823	to 1551483	2254456
							ESCO_003940
EtpG (glutathione S-transferase)	298625 to 297739	2109705 to 2108806	2276410 to 2277314	3175327 to 3174437	244610 to 243735	1550270 to 1549395	2255743 to 2256647
							ESCO_003941
EtpK (γ-glutamyl cyclo transferase)	293170 to 294157	2103671 to 2104871	2281747 to 2280743	3168563 to 3169394	238798 to 239801	1544342 to 1545461	2261031 to 2260005
							ESCO_003942
EtpP (nonribosomal peptide synthetase)	285789 to 292009	2096364 to 2102596	2289078 to 2282852	3161192 to 3167407	231491 to 237723	1537035 to 1543267	2268362 to 2262136
							ESCO_003943
EtpC (cytochrome P450 mono-oxygenase)	283494 to 282567	2094052 to 2093122	2291375 to 2292302	3158664 to 3157570	229174 to 228244	1534726 to 1533797	2270659 to 2271586
							ESCO_003944
EtpI (PLP-dependent carbon-sulphur bond lyase)	280748 to 282358	2091214 to 2092868	2294266 to 2292511	3155443 to 3157194	226286 to 227990	1531912 to 1533543	2273419 to 2271795
							ESCO_003945
EtpN (methyl transferase)	280119 to 279055	2090539 to 2089447	2294895 to 2295970	3154660 to 3153439	225611 to 224519	1531236 to 1530144	2274048 to 2275127
							ESCO_003946

Supplementary Table 5 | Position of genes and predicted function for the ETP BGCs in the genomes of sequenced *Escovopsis* strains. The annotated BGC from strain G was given the accession number TPA: BK010420.

Escovopsis strain	**Strain G**	*C. virescens* ETP Cluster	E value	Identities
**Contig**	LGSR01000002.1	KF496217		
**Gene name (predicted function)**	**Strain G position**	**Gene Name**		
	**Gene Identifier**	**Gene Identifier**		
EtpT (thioredoxin reductase)	2232345 to 2231095	ChaT	8e-125, 8e-125, 8e-125	130/165 (79%), 83/118 (70%), 11/22 (50%)
	ESCO_003933			
EtpE (cytochrome P450 mono-oxygenase)	2233958 to 2235711	ChaE	8e-92, 8e-92, 8e-92	140/389 (36%), 57/86 (66%), 38/52 (73%)
	ESCO_003934			
EtpM (methyl transferase)	2236079 to 2237369	ChaM	8e-151	224/351 (64%)
	ESCO_003935			
EtpA (ABC transporter)	2242965 to 2238451	ChaA	0.0, 0.0, 0.0, 1e-110, 1e-110	387/621 (62%), 77/133 (58%), 26/38 (68%), 142/185 (77%), 59/107 (55%)
	ESCO_003936			
EtpB (cytochrome P450 mono-oxygenase)	2246484 to 2244404	ChaB	4e-117, 4e-117	128/196 (65%), 82/112 (73%)
	ESCO_003937			
EtpG (glutathione S-transferase)	2255743 to 2256647	ChaG	1e-90, 1e-90	99/146 (68%), 62/78 (79%)
	ESCO_003941			
EtpK (γ-glutamyl cyclo transferase)	2261031 to 2260005	ChaK	1e-38	61/91 (67%)
	ESCO_003942			
EtpP	2268362 to 2262136	ChaP	0.0	1064/2132

(nonribosomal peptide synthetase)	ESCO_003943			(50%)
EtpC (cytochrome P450 mono-oxygenase)	2270659 to 2271586	ChaC	3e-45, 1e-29, 1e-29	87/204 (43%), 30/32 (94%), 27/34 (79%)
	ESCO_003944			
EtpI (PLP-dependent carbon-sulphur bond lyase)	2273419 to 2271795	ChaI	2e-140, 2e-140, 2e-140	86/129 (67%), 92/154 (60%), 98/164 (60%)
	ESCO_003945			
EtpN (methyl transferase)	2274048 to 2275127	ChaN	2e-98, 2e-98	142/268 (53%), 38/59 (64%)
	ESCO_003946			

Supplementary Table 6 | TBLASTN [13] analysis of the nucleotide sequences of the ETP BGC from *C. virescens* [3] and the proteins encoded by ETP BGC from the reference genome of *Escovopsis* strain G. Similarity was assessed according to E value and shared amino acid identities.

***Escovopsis* strain**	Strain G	Strain A	E value	Identities	Strain B	E value	Identities	Strain C	E value	Identities	Strain D	E value	Identities	Strain E	E value	Identities	Strain F	E value	Identities
**Contig**	LGSR01000002.1	SC05			SC07			SC05			SC05			SC024			SC10		
**Gene name (predicted function)**	**Strain G**<br>**Gene Identifier**	**Strain A position**			**Strain B position**			**Strain C position**			**Strain D position**			**Strain E position**			**Strain F position**		
EtpT (thioredoxin reductase)	2232345 to 2231095<br>ESCO_003933	324397 to 325642	5e-109, 2e-84, 3e-16	163/175 (93%), 145/158 (92%), 33/33 (100%)	2133685 to 2134931	3e-170, 3e-170, 3e-170	159/163 (98%), 139/143 (97%), 32/33 (97%)	2252891 to 2251641	4e-174, 4e-174, 4e-174	164/169 (97%), 142/143 (99%), 33/33 (100%)	3202227 to 3203420	3e-150, 3e-150, 3e-150	143/161 (89%), 123/143 (86%), 28/34 (82%)	268585 to 269831	2e-170, 2e-170, 2e-170	159/163 (98%), 139/143 (97%), 32/33 (97%)	1574269 to 1575515	3e-170, 3e-170, 3e-170	159/163 (98%), 139/143 (97%), 32/33 (97%)
EtpE (cytochrome P450 mono-oxygenase)	2233958 to 2235711<br>ESCO_003934	322606 to 320855	0.0	524/547 (96%)	2131923 to 2130141	0.0, 0.0	375/392 (96%), 129/136 (95%)	2254504 to 2256257	0.0, 0.0	390/390 (100%), 136/136 (100%)	3199415 to 3197632	0.0, 3e-77	336/396 (85%), 114/147 (78%)	266791 to 265009	0.0, 0.0	376/392 (96%), 128/133 (96%)	1572507 to 1570725	0.0, 0.0	376/392 (96%), 129/136 (95%)
EtpM (methyl transferase)	2236079 to 2237369<br>ESCO_003935	320487 to 319147	0.0	399/446 (89%)	2129695 to 2128413	0.0, 6e-33	297/331 (90%), 69/73 (95%)	2256625 to 2257915	0.0, 4e-34	331/331 (100%), 72/73 (99%)	3197206 to 3195983	2e-172	278/388 (72%)	264563 to 263276	0.0, 2e-32	297/331 (90%), 69/73 (95%)	1570279 to 1568992	0.0, 2e-32	297/331 (90%), 69/73 (95%)
EtpA (ABC transporter)	2242965 to 2238451<br>ESCO_003936	313586 to 318100	0.0, 5e-175, 4e-141, 4e-141, 4e-141, 1e-51, 1e-51,	403/442 (91%), 300/343 (87%), 231/327 (71%), 47/50 (94%), 20/20 (100%), 79/100 (79%), 41/47 (87%)	2122708 to 2127241	0.0, 0.0, 0.0, 0.0, 0.0, 0.0, 0.0, 0.0, 0.0, 3e-08	398/445 (89%), 183/184 (99%), 129/134 (96%), 131/200 (66%), 91/96 (95%), 66/88 (75%), 30/31 (97%), 26/32 (81%), 12/16 (75%), 23/30 (77%)	2263511 to 2258997	0.0, 1e-172, 1e-172, 8e-36, 8e-36, 2e-31, 2e-31	640/721 (89%), 207/228 (91%), 96/96 (100%), 39/39 (100%), 81/100 (81%), 49/52 (94%), 20/20 (100%)	3190085 to 3194823	0.0, 2e-146, 2e-146, 4e-135, 4e-135, 4e-41, 4e-41, 6e-08	390/505 (77%), 181/187 (97%), 92/112 (82%), 121/132 (92%), 133/199 (67%), 60/119 (50%), 39/39 (100%), 20/21 (95%)	257540 to 262104	0.0, 0.0, 0.0, 0.0, 0.0, 0.0, 0.0, 0.0, 0.0, 3e-08	397/445 (89%), 183/184 (99%), 128/134 (96%), 131/200 (66%), 92/96 (96%), 66/88 (75%), 30/31 (97%), 26/32 (81%), 12/16 (75%), 23/30 (77%)	1563285 to 1567820	0.0, 0.0, 0.0, 8e-173, 1e-14, 1e-14, 9e-14, 3e-08	397/445 (89%), 129/134 (96%), 131/200 (66%), 329/489 (67%), 26/32 (81%), 12/16 (75%), 30/31 (97%), 23/30 (77%)
EtpB (cytochrome P450 mono-	2246484 to 2244404	310114 to	0.0, 0.0	377/440 (86%),	2119099 to	2e-	203/222	2267129 to	0.0, 0.0,	244/266 (92%),	3186108 to	2e-141,	198/236 (84%),	254012 to	3e-147,	203/222 (91%),	1559626 to	9e-145,	200/219 (91%),

oxygenase)	ESCO_003937	312202		166/234 (71%)	2121302	146,<br>4e-83<br>4e-83<br>3e-76	(91%),<br>82/100 (82%),<br>71/114 (62%),<br>128/134 (96%)	2264955	0.0, 2e-79, 0.001	141/179 (79%), 27/28 (96%), 138/144 (96%), 11/13 (85%)	3188282	1e-70, 5e-55 5e-55 6e-21 6e-21	120/137 (88%), 64/97 (66%), 49/79 (62%), 26/30 (87%), 25/42 (60%)	256130	2e-82 2e-82 6e-77	82/100 (82%), 70/101 (69%), 128/134 (96%)	1561876	2e-82 2e-82 7e-77 2e-10	82/100 (82%), 70/101 (69%), 128/134 (96%), 27/49 (55%)
ORF (unknown)	2248934 to 2248014<br>ESCO_003938	306645 to 307583	3e-115	241/312 (77%)	2116530 to 2117474	9e-82	184/318 (58%)	2269582 to 2268662	4e-124	241/306 (79%)	3183054 to 3184079	2e-64 5e-08	136/204 (67%), 15/20 (75%)	251449 to 252387	6e-85	190/316 (60%)	1557063 to 1558001	6e-85	190/316 (60%)
ORF (unknown)	2249158 to 2249615<br>ESCO_003939	306421 to 306002	5e-35, 0.058	54/55 (98%), 6/7 (86%)	2116300 to 2116082	1e-31	51/55 (93%)	2269806 to 2270264	9e-36	55/55 (100%)	3182743 to 3182519	1e-15	34/56 (61%)	251219 to 251001	1e-31	51/55 (93%)	1556833 to 1556615	1e-31	51/55 (93%)
ORF (unknown)	2253731 to 2254456<br>ESCO_003940	301951 to 301202	2e-64 8e-59	90/92 (98%), 86/87 (99%)	2111637 to 2110942	1e-102	156/233 (67%)	2274390 to 2275115	2e-67 1e-59	93/93 (100%), 87/87 (100%)	3177538 to 3176795	4e-46 6e-44	69/88 (78%), 67/89 (75%)	246518 to 245823	1e-102	156/233 (67%)	1552178 to 1551483	1e-102	156/233 (67%)
EtpG (glutathione S-transferase)	2255743 to 2256647<br>ESCO_003941	298625 to 297739	2e-129 2e-129 2e-129	124/126 (98%), 86/90 (96%), 24/25 (96%)	2109705 to 2108806	4e-159	225/299 (75%)	2276410 to 2277314	1e-133, 1e-133	150/184 (82%), 89/94 (95%)	3175327 to 3174437	7e-144	205/296 (69%)	244610 to 243735	3e-159	224/291 (77%)	1550270 to 1549395	6e-160	225/291 (77%)
EtpK (γ-glutamyl cyclo transferase)	2261031 to 2260005<br>ESCO_003942	293170 to 294157	3e-63, 5e-09, 1e-08	89/90 (99%), 16/16 (100%), 18/22 (82%)	2103671 to 2104871	2e-61 6e-09 2e-05	88/90 (98%), 16/16 (100%), 13/18 (72%)	2281747 to 2280743	3e-63 1e-09 9e-09	89/90 (99%), 19/22 (86%), 16/16 (100%)	3168563 to 3169394	8e-58 3e-05	82/90 (91%), 12/14 (86%)	238798 to 239801	4e-62 5e-09 2e-05	88/90 (98%), 16/16 (100%), 13/18 (72%)	1544342 to 1545461	1e-61 6e-09 1e-05	88/90 (98%), 16/16 (100%), 13/22 (59%)
EtpP (nonribosomal peptide synthetase)	2268362 to 2262136<br>ESCO_003943	285789 to 292009	0.0, 0.0	1018/1109 (92%), 900/942 (96%)	2096364 to 2102596	0.0, 0.0	954/1166 (82%), 817/879 (93%)	2289078 to 2282852	0.0, 0.0	1038/1109 (94%), 912/942 (97%)	3161192 to 3167407	0.0	1484/2088 (71%)	231491 to 237723	0.0, 0.0	955/1166 (82%), 821/888 (92%)	1537035 to 1543267	0.0, 0.0	956/1166 (82%), 816/879 (93%)
EtpC (cytochrome P450 mono-oxygenase)	2270659 to 2271586<br>ESCO_003944	283494 to 282567	1e-132<br>1e-132, 1e-132, 1e-132	107/109 (98%), 74/76 (97%), 41/47 (87%), 21/24 (88%)	2094052 to 2093122	1e-112, 9e-25 2e-06	171/242 (71%), 39/47 (83%), 13/17 (76%)	2291375 to 2292302	6e-136, 6e-136, 6e-136, 6e-136	107/109 (98%), 77/82 (94%), 46/47 (98%), 21/24 (88%)	3158664 to 3157570	4e-122	186/332 (56%)	229174 to 228244	1e-112, 9e-25 2e-06	171/242 (71%), 39/47 (83%), 13/17 (76%)	1534726 to 1533797	6e-145, 8e-06	207/313 (66%), 13/15 (87%)
EtpI (PLP-	2273419 to	280748	4e-174,	153/154	2091214	0.0,	293/379	2294266	6e-	154/154	3155443	0.0	300/564	226286	1e-	135/156	1531912	0.0	407/543

dependent carbon-sulphur bond lyase)	2271795<br>ESCO_003945	to 282358	4e-174, 3e-87	(99%), 176/178 (99%), 127/129 (98%)	to 2092868	1e-76	(77%), 114/129 (88%)	to 2292511	107, 2e-99 2e-87	(100%), 178/178 (100%), 129/129 (100%)	to 3157194		(53%)	to 227990	92, 1e-85 2e-76	(87%), 159/180 (88%), 114/129 (88%)	to 1533543		(75%)
EtpN (methyl transferase)	2274048 to 2275127<br>ESCO_003946	280119 to 279055	2e-158, 2e-158, 2e-158	155/155 (100%), 92/119 (77%), 38/41 (93%)	2090539 to 2089447	1e-138, 1e-138, 1e-138, 1e-138	148/155 (95%), 50/52 (96%), 33/42 (79%), 24/29 (83%)	2294895 to 2295970	5e-162, 5e-162	196/223 (88%), 93/116 (80%)	3154660 to 3153439	3e-116, 3e-116, 1e-19	126/156 (81%), 79/127 (62%), 32/41 (78%)	225611 to 224519	1e-138, 1e-138, 1e-138, 1e-138	148/155 (95%), 50/52 (96%), 33/42 (79%), 24/29 (83%)	1531236 to 1530144	2e-144, 2e-144, 2e-144, 2e-144	147/155 (95%), 62/70 (89%), 33/42 (79%), 24/29 (83%)

Supplementary Table 7 | BLASTX [13] analysis of the proteins encoded by the ETP BGC from the reference genome of *Escovopsis* strain G and the nucleotide sequences of the ETP BGC from *Escovopsis* strains A-F. Similarity was assessed according to E value and shared amino acid identities.

***Escovopsis* strain**	Strain A	Strain B	Strain C	Strain D	Strain E	Strain F	Strain G
**Contig**	SC08	SC03	SC06	SC10	SC020	SC02	LGSR01000019.1
**Gene name (predicted function)**	**Strain A**	**Strain B**	**Strain C**	**Strain D**	**Strain E**	**Strain F**	**Strain G**<br>**Gene Identifier**
ORF (unknown)	1337019 to 1337533	3152647 to 3153166	130574 to 130060	108840 to 108284	278762 to 278244	2895728 to 2896247	910902 to 911416<br>ESCO_005789
ORF (unknown)	1339235 - 1339769	n/a	128398-127966	n/a	n/a	n/a	913079 to 913511<br>ESCO_005790
ORF (unknown)	1342103 to 1340090	3157964 to 3155949	125471 to 127475	105348 to 107505	273445 to 275460	2901048 to 2899033	916006 to 914002<br>ESCO_005791
EmoH (decarboxylase)	1343633 to 1343074	3160231 to 3159620	124163 to 124723	102658 to 103255	271134 to 271789	2903362 to 2902705	917314 to 916754<br>ESCO_005792
EmoM (mono-oxygenase)	1344567 to 1345240	3161185 to 3161851	123221 to 122548	101299 to 100640	270187 to 269522	2904316 to 2904982	918256 to 918929<br>ESCO_005793
EmoF (hydrolase)	1346355 to 1345457	3162974 to 3162072	121433 to 122331	99512 to 100439	268399 to 269301	2906105 to 2905203	920044 to 919146<br>ESCO_005794
EmoG (polyketide synthase)	1347475 to 1352937	3164091 to 3169553	120312 to 114839	98335 to 92840	267282 to ? (stop codon unclear)	2907222 to 2912684	921165 to 926638<br>ESCO_005795
ORF (unknown)	1358116 to	3174683 to	109708 to	87806 to ? (stop	258204 to	2917808 to	931768 to 930709

	1357058	3173618	110768	codon unclear)	259269	2916743	ESCO_005796

Supplementary Table 8 | Position of genes and predicted function for the emodin (8) BGC in the genomes of sequenced *Escovopsis* strains. The annotated cluster from strain G was given the accession number TPA: BK010419.

Escovopsis **strain**	Strain G	*A. nidulans* monodictyphenone Cluster	E value	Identities
**Contig**	LGSR01000019.1	chromosome: CADRE2:<br>VIII:4354659:4456428:1		
**Gene name (predicted function)**	**Strain G position**<br>**Gene Identifier**	**Gene Name**<br>**Gene Identifier**		
EmoH (decarboxylase)	917314 to 916754<br>ESCO_005792	MdpH<br>ANIA_10022	1e-58, 0.36	77/143 (54%), 24/126 (19%)
EmoF (hydrolase)	920044 to 919146<br>ESCO_005794	MdpF<br>ANIA_00149	1e-118	163/271 (60%)
EmoG (polyketide synthase)	921165 to 926638<br>ESCO_005795	MdpG<br>ANIA_00150	0.0, 4e-30	910/1582 (58%), 56/74 (76%)

Supplementary Table 9 | BLASTP [13] analysis of the proteins encoded by the monodictyphenone BGC, that makes emodin, from *A. nidulans* [4] and the reference genome of *Escovopsis* strain G. Similarity was assessed according to E value and shared amino acid identities.

***Escovopsis* strain**	Strain G	Strain A	E value	Identities	Strain B	E value	Identities	Strain C	E value	Identities	Strain D	E value	Identities	Strain E	E value	Identities	Strain F	E value	Identities
**Contig**	LGSR01000019.1	SC08			SC03			SC06			SC10			SC020			SC02		
**Gene name (predicted function)**	**Strain G**<br>**Gene Identifier**	**Strain A position**			**Strain B position**			**Strain C position**			**Strain D position**			**Strain E position**			**Strain F position**		
ORF (unknown)	910902 to 911416<br>ESCO_005789	1337019 to 1337533	5e-73, 5e-73	102/105 (97%), 44/46 (96%)	3152647 to 3153166	8e-72, 1e-15	94/103 (91%), 43/46 (93%)	130574 to 130060	3e-78, 9e-17	102/105 (97%), 45/46 (98%)	108840 to 108284	5e-51, 3e-06	71/111 (64%), 14/29 (48%)	278762 to 278244	3e-88	137/172 (80%)	2895728 to 2896247	3e-88	137/172 (80%)
ORF (unknown)	913079 to 913511<br>ESCO_005790	1339235-1339769	9e-37, 2e-32	58/76 (76%), 50/51 (98%)	n/a	n/a	n/a	128398-127966	5e-58, 5e-58	59/63 (94%), 51/51 (100%)	n/a	n/a	n/a	n/a	n/a	n/a	n/a	n/a	n/a
ORF (unknown)	916006 to 914002<br>ESCO_005791	1342103 to 1340090	0.0, 0.0, 0.0	311/314 (99%), 138/176 (78%), 111/112 (99%)	3157964 to 3155949	0.0,	507/651 (78%)	125471 to 127475	0.0, 0.0, 0.0	313/314 (99%), 138/175 (79%), 112/112 (100%)	105348 to 107505	0.0, 0.0, 0.0, 0.0	168/246 (68%), 153/203 (75%), 84/116 (72%), 23/48 (48%)	273445 to 275460	0.0	507/651 (78%)	2901048 to 2899033	0.0	507/651 (78%)
EmoH (decarboxylase)	917314 to 916754<br>ESCO_005792	1343633 to 1343074	2e-90, 7e-11	124/152 (82%), 20/20 (100%)	3160231 to 3159620	4e-92	134/203 (66%)	124163 to 124723	8e-47, 4e-44, 2e-11	65/73 (89%), 64/89 (72%), 20/20 (100%)	102658 to 103255	5e-42, 1e-34	64/105 (61%), 51/78 (65%)	271134 to 271789	4e-83, 7e-11	115/152 (76%), 20/20 (100%)	2903362 to 2902705	4e-83, 7e-11	115/152 (76%), 20/20 (100%)
EmoM (mono-oxygenase)	918256 to 918929<br>ESCO_005793	1344567 to 1345240	4e-97, 2e-24	151/152 (99%), 41/41 (100%)	3161185 to 3161851	2e-68, 2e-21	145/173 (84%), 37/41 (90%)	123221 to 122548	6e-98, 2e-24	152/152 (100%), 41/41 (100%)	101299 to 100640	1e-103	142/219 (65%)	270187 to 269522	9e-89	177/221 (80%)	2904316 to 2904982	9e-89	177/221 (80%)
EmoF (hydrolase)	920044 to 919146<br>ESCO_005794	1346355 to 1345457	0.0, 6e-07	254/259 (98%), 15/15 (100%)	3162974 to 3162072	0.0	258/300 (86%)	121433 to 122331	0.0, 5e-07	258/259 (99%), 15/15 (100%)	99512 to 100439	4e-144, 2e-05	204/262 (78%), 11/15 (73%)	268399 to 269301	0.0	258/300 (86%)	2906105 to 2905203	0.0	258/300 (86%)
EmoG (polyketide synthase)	921165 to 926638<br>ESCO_005795	1347475 to 1352937	0.0, 9e-134, 2e-30,	1198/1258 (95%), 210/212 (99%), 56/57	3164091 to 3169553	0.0, 7e-122, 9e-39,	1127/1266 (89%), 191/204 (94%), 70/76	120312 to 114839	0.0, 4e-135, 9e-31,2e-	1218/1258 (97%), 212/212 (100%), 57/57	98335 to 92840	0.0, 4e-104, 7e-21,	971/1288 (75%), 162/212 (76%), 39/46	267282 to ? (stop codon unclear)	0.0, 2e-120, 3e-	1124/1266 (89%), 190/204 (93%), 43/48	2907222 to 2912684	0.0, 7e-117, 1e-	1124/1266 (89%), 190/204 (93%), 43/48

			1e-22	(98%), 54/100 (54%)		3e-23,	(92%), 43/48 (90%)		23	(100%), 55/100 (55%)		2e-12	(85%), 26/47 (55%)	262078 was assumed	23	(90%)		20	(90%)
ORF (unknown)	931768 to 930709<br>ESCO_005796	1358116 to 1357058	6e-143, 2e-73	191/201 (95%), 106/111 (95%)	3174683 to 3173618	2e-137, 4e-71	182/196 (93%), 100/111 (90%)	109708 to 110768	0.0	295/322 (92%)	87806 to ? (stop codon unclear) 88799 was assumed	4e-111, 5e-60	163/210 (78%), 83/107 (78%)	258204 to 259269	4e-138, 4e-71	183/196 (93%), 100/111 (90%)	2917808 to 2916743	4e-138, 4e-71	183/196 (93%), 100/111 (90%)

Supplementary Table 10 | BLASTX [13] analysis of the proteins encoded by the emodin BGC from the reference genome of *Escovopsis* strain G and the nucleotide sequences of the emodin BGC from *Escovopsis* strains A-F. Similarity was assessed according to E value and shared amino acid identities.

**Strain_sequence**	**A_*tef1***	**B_*tef1***	**C_*tef1***	**D_*tef1***	**E_*tef1***	**F_*tef1***	**G_*tef1***
**A_*tef1***	100.00	96.70	98.29	88.65	96.70	96.70	98.28
**B_*tef1***	96.70	100.00	96.31	88.26	100.00	100.00	96.31
**C_*tef1***	98.29	96.31	100.00	88.52	96.31	96.31	100.00
**D_*tef1***	88.65	88.26	88.52	100.00	88.26	88.26	88.52
**E_*tef1***	96.70	100.00	96.31	88.26	100.00	100.00	96.31
**F_*tef1***	96.70	100.00	96.31	88.26	100.00	100.00	96.31
**G_*tef1***	98.28	96.31	100.00	88.52	96.31	96.31	100.00
**Strain_sequence**	**A_ITS**	**B_ITS**	**C_ITS**	**D_ITS**	**E_ITS**	**F_ITS**	
**A_ITS**	100.00	95.80	99.27	86.35	95.80	95.80	
**B_ITS**	95.80	100.00	95.80	86.90	100.00	100.00	
**C_ITS**	99.27	95.80	100.00	86.35	95.80	95.80	
**D_ITS**	86.35	86.90	86.35	100.00	86.90	86.90	
**E_ITS**	95.80	100.00	95.80	86.90	100.00	100.00	
**F_ITS**	95.80	100.00	95.80	86.90	100.00	100.00	

Supplementary Table 11 | Percentage Identity Matrix for partial alignment of the nucleotide sequences for the elongation factor 1-alpha gene (*tef1*) and for the internal transcriber spacer (ITS).

Name	Sequence	Reference
**18S 1A**	AAC CTG GTT GAT CCT GCC AGT	**14**
**18S 564**	GGC ACC AGA CTT GCC CTC	**14**
**ITS1**	TCC GTA GGT GAA CCT GCG G	**7**
**ITS4**	TCC TCC GCT TAT TGA TAT GC	**7**

Supplementary Table 12 | Oligonucleotides used in this work.

Analyte	Precursor ion (*m*/*z*)	Cone voltage (V)	Product ion (*m*/*z*)	Collision energy (V)	Dwell time (msec)
shearinine D (**2**)	600.4	40	210.2	50	25
			238.2	38	25
			484.3	30	25
			542.4	18	25
melinacidin IV (**1**)	729.1	36	185.1	34	25
			282.1	28	25
			300.2	34	25
			647.2	18	25
yohimbine	355.3	98	162.1	30	22
			194.2	30	22
			212.2	22	22
			224.2	22	22

Supplementary Table 13 | Mass transitions used in the MRM method for MS/MS based quantitation of melinacidin IV (1) and shearinine D (2); internal standard: yohimbine.

Supplementary Note 1 | Chemical characterization of Melinacidin IV (**1**): ^{1}H NMR (DMSO-d_6, 400 MHz) *δ* 7.71 (2H, d, *J* = 7.6 Hz), 7.03 (2H, td, *J* = 7.6 Hz, *J* = 0.8 Hz), 6.71 (2H, s), 6.65 (dd, *J* = 7.6 Hz, *J* = 0.8 Hz), 6.54 (2H, d, *J* = 7.8 Hz), 6.51 (2H, d, *J* = 4.2 Hz), 5.82 (2H, m), 5.26 (2H, s), 4.88 (2H, s), 4.21 (2H, dd, *J* = 12.6 Hz, *J* =3.0), 4.06 (2H, d, *J* = 12.6 Hz, *J* =4.3), 3.00 (6H, s); HR-ESI-MS: *m*/*z* calculated for: $C_{30}H_{29}N_6O_8S_4$: 729.0924; observed: 729.0937 $[M+H]^+$: Δ = 1.78 ppm; MS^2: 729.1>665.1, 729.1>647.1, 729.1>481.1; UV/Vis (MeOH): λ_{max} = 210, 240, 301 nm. Analytical data are in full agreement with literature values [15].

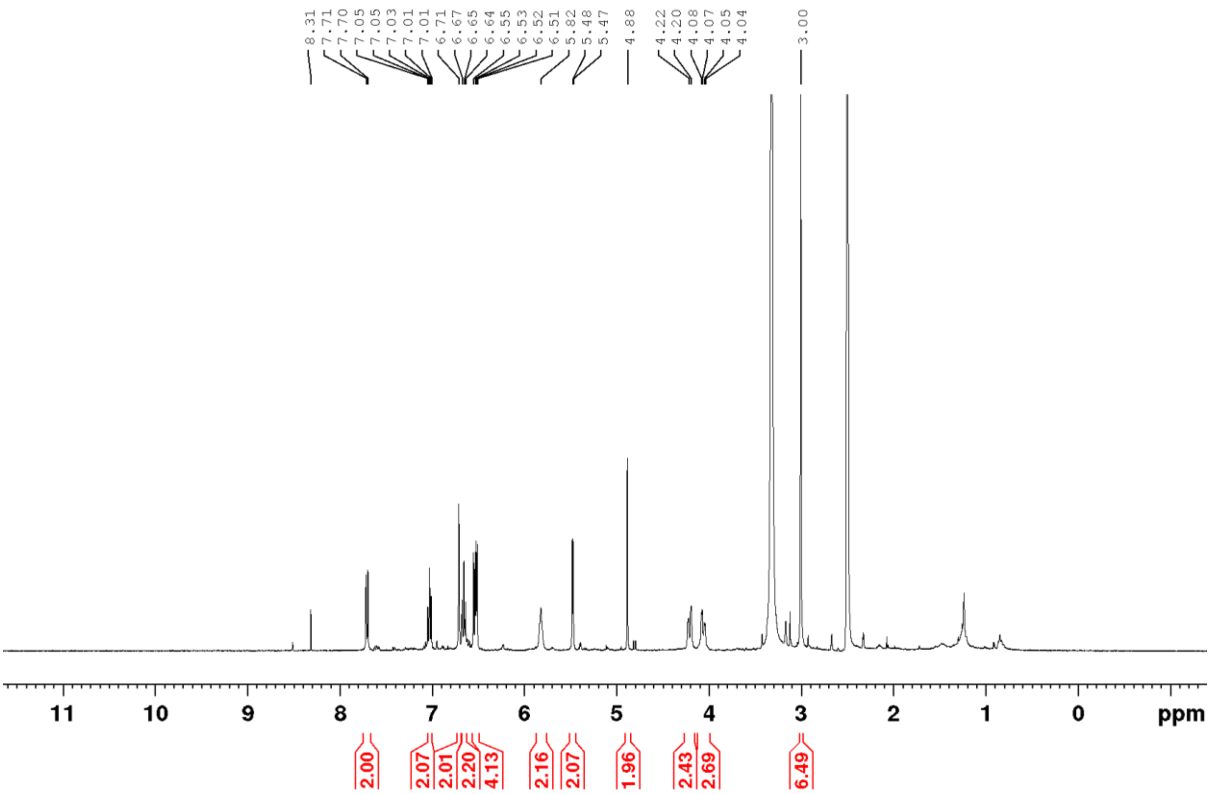

Supplementary Figure 14 | ^{1}H NMR spectrum (DMSO-d_6, 400 MHz) of compound 1

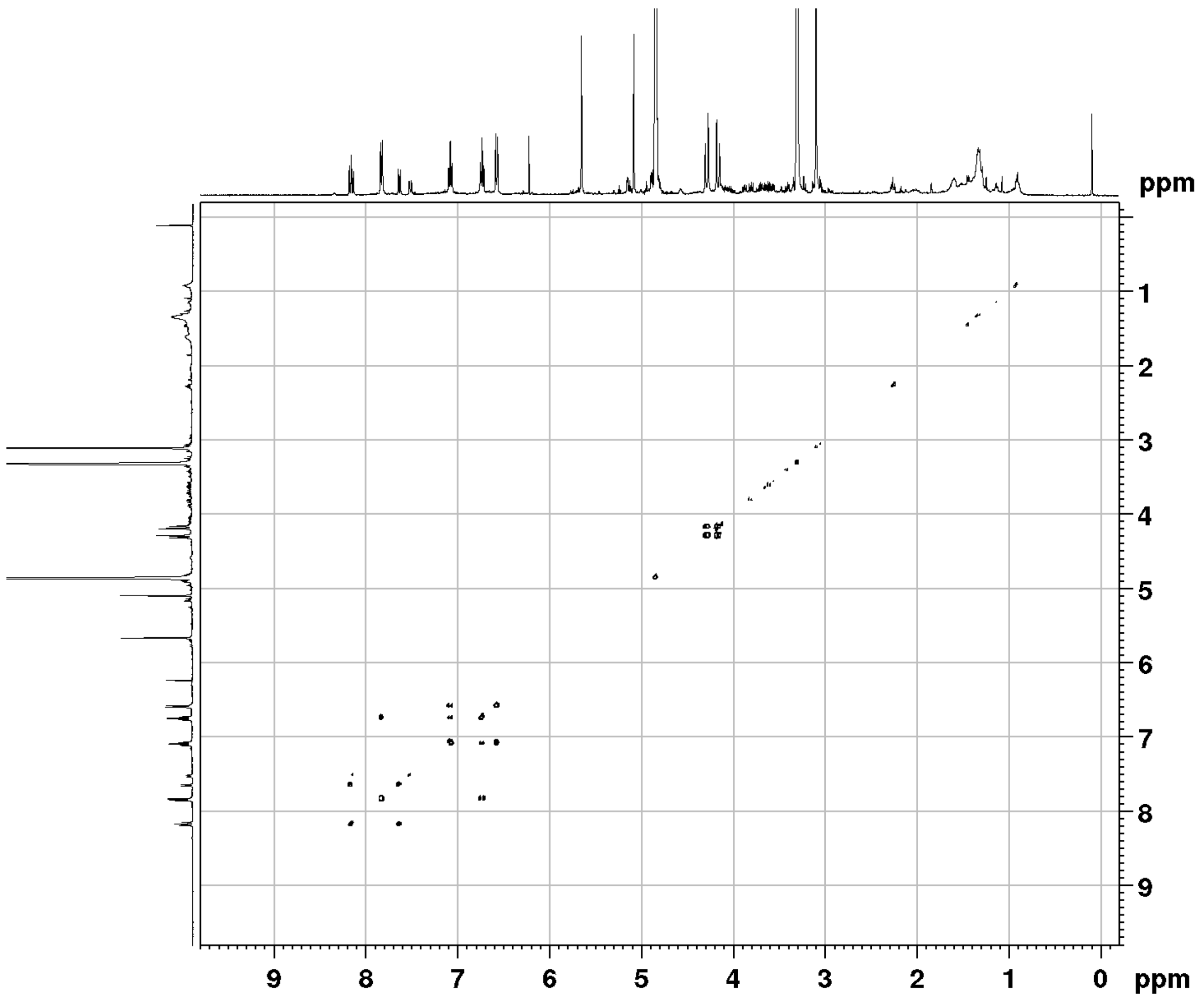

Supplementary Figure 15 | COSY spectrum (MeOD, 400 MHz) of compound 1

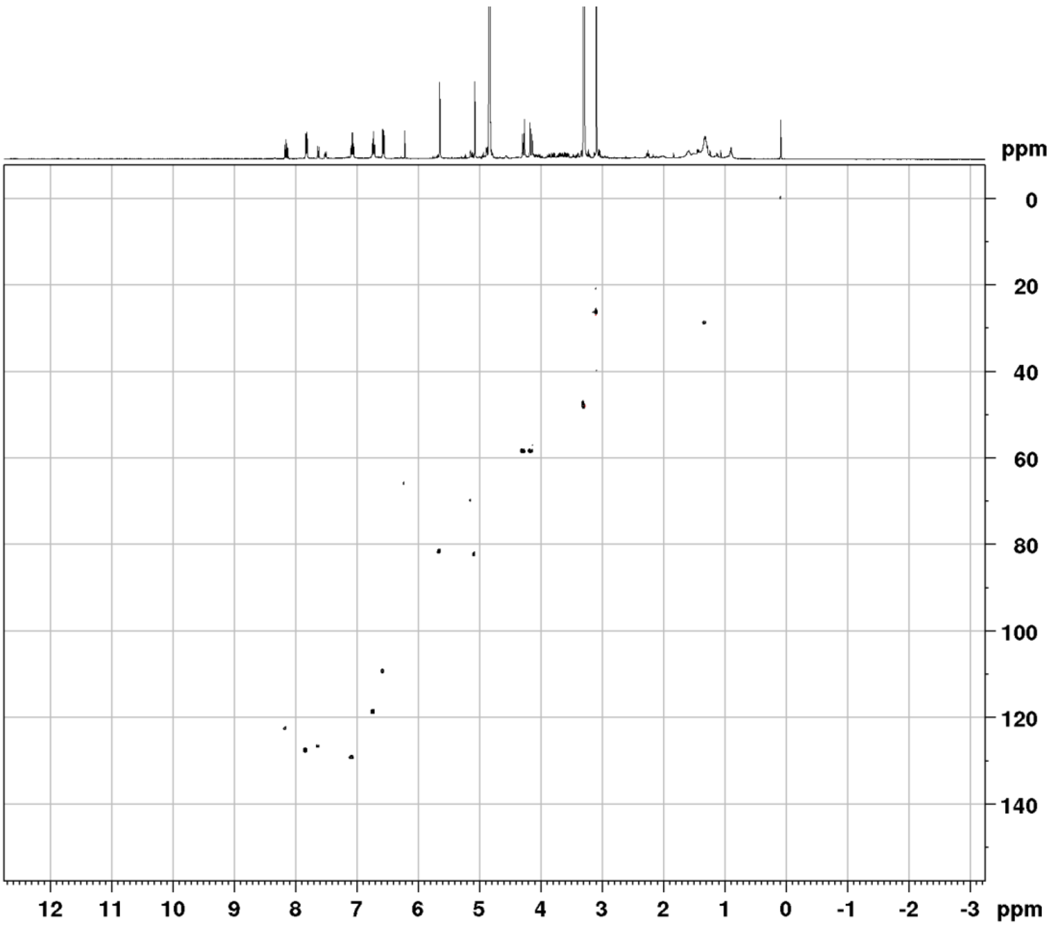

Supplementary Figure 16 | HSQC spectrum (MeOD, 400 MHz) of compound 1

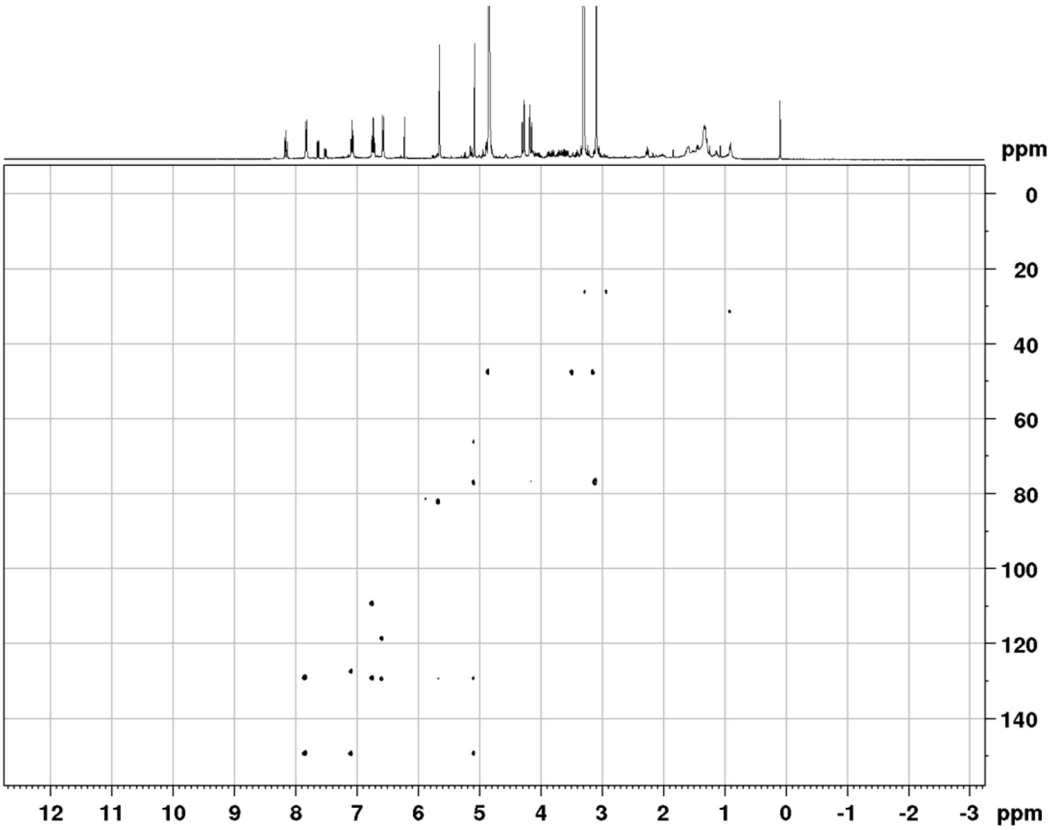

Supplementary Figure 17 | HMBC spectrum (MeOD, 400 MHz) of compound 1

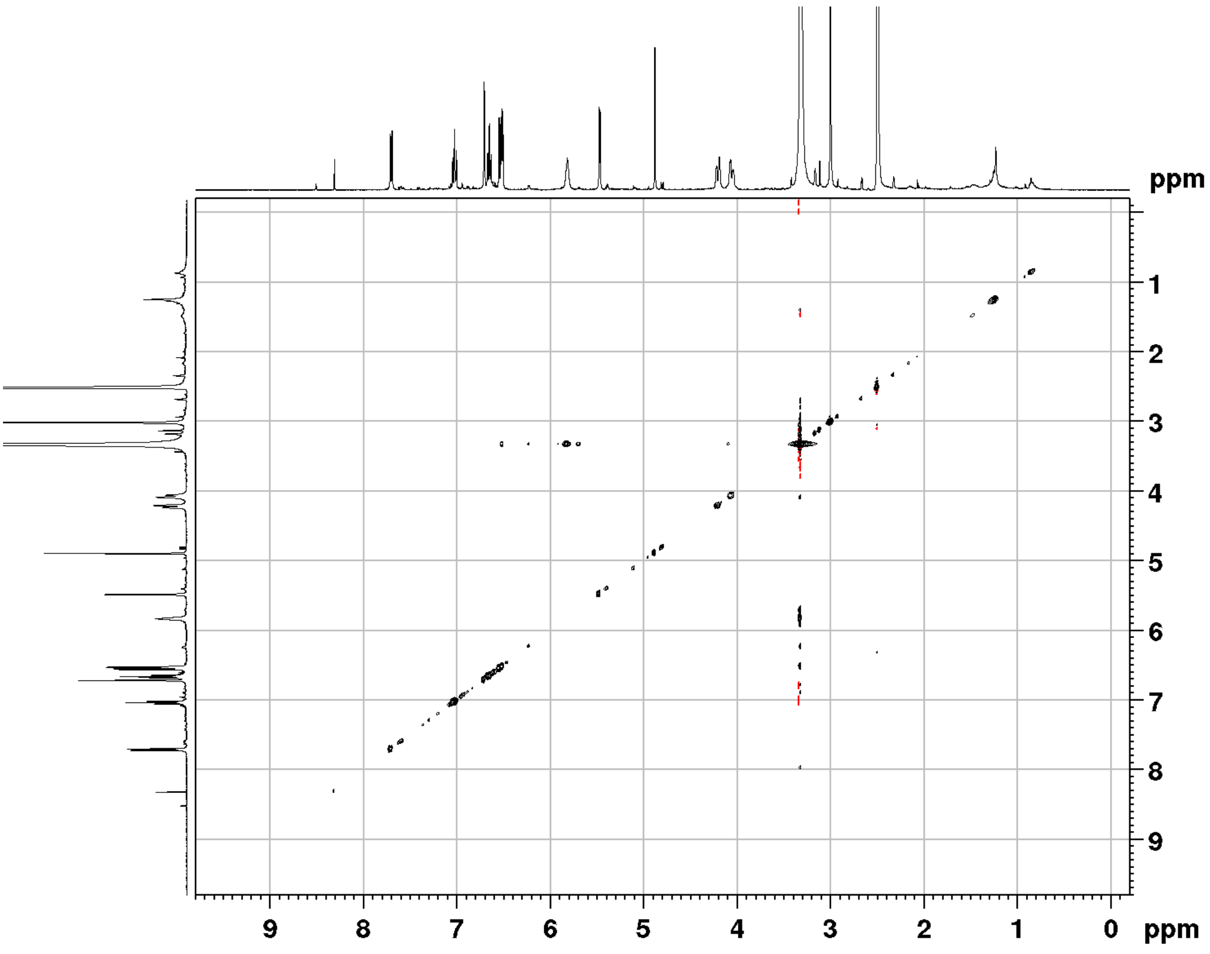

Supplementary Figure 18 | NOESY NMR spectrum (DMSO-d_6, 400 MHz) of compound 1

Supplementary Note 2 | Chemical characterization of shearinine D (**2**): ^{1}H NMR ($CDCl_3$, 400 MHz) δ 7.47 (1H, s), 7.33 (1H, m), 5.95 (1H, d, J = 2.9 Hz), 5.83 (1H, m), 4.95 (1H, m) 4.30 (1H, d, J = 1.2 Hz), 2.63-2.85 (5H, m), 2.42 (1H, dd, J = 13.0 Hz, J = 10.6 Hz), 1.95-2.08 (4H, m), 1.76-1.91 (2H, m) 1.52 (3H, s), 1.48 (3H, s) 1.43 (3H, s), 1.37 (3H, s), 1.34 (3H, s), 1.33 (3H, s), 1.22 (3H, s), 1.17 (3H, s), 1.10 (3H, s); ^{13}C NMR ($CDCl_3$, 100 MHz) δ 197.0, 169.5, 153.8, 141.0, 138.6, 135.1, 131.8, 126.8, 120.9, 117.7, 117.5, 113.9, 104.3, 102.6, 88.0, 78.8, 77.7, 76.5, 73.8, 72.4, 60.1, 51.7, 48.5, 39.9, 33.9, 31.9, 30.0, 29.8, 28.8, 28.2, 27.5, 27.0, 23.6, 23.1, 23.0, 21.1, 16.1 ppm; HR-ESI-MS: m/z calculated for: $C_{37}H_{46}NO_6$: 600.3320; observed: 600.3340 $[M+H]^+$: Δ = 3.33 ppm; UV/Vis (MeOH): λ_{max} = 210, 236, 258, 330 nm; $[\alpha]_D$ +26 °, (c = 0.05, $CHCl_3$). Analytical data are in full agreement with literature values [16].

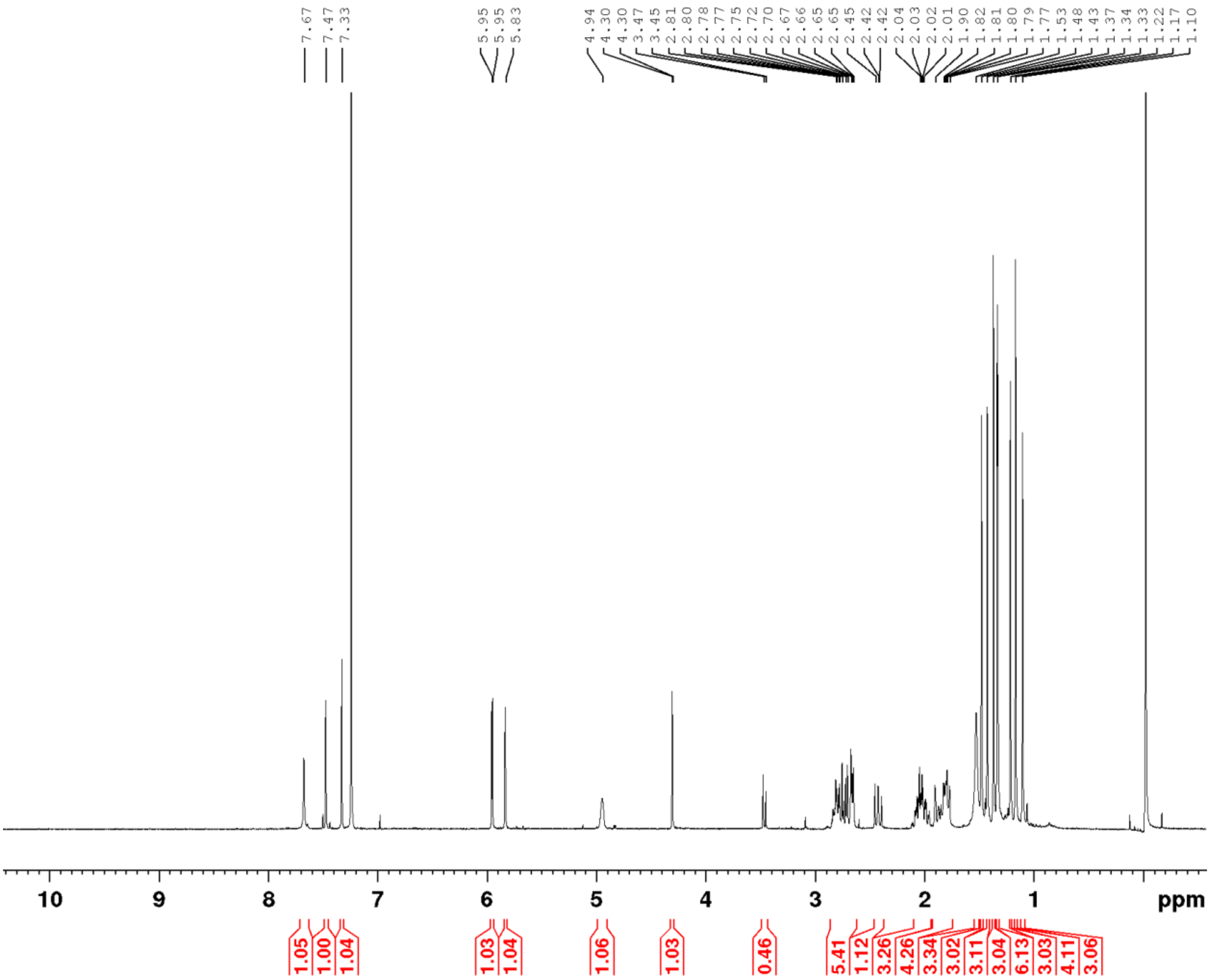

Supplementary Figure 19 | ^{1}H NMR spectrum ($CDCl_3$, 400 MHz) of compound 2

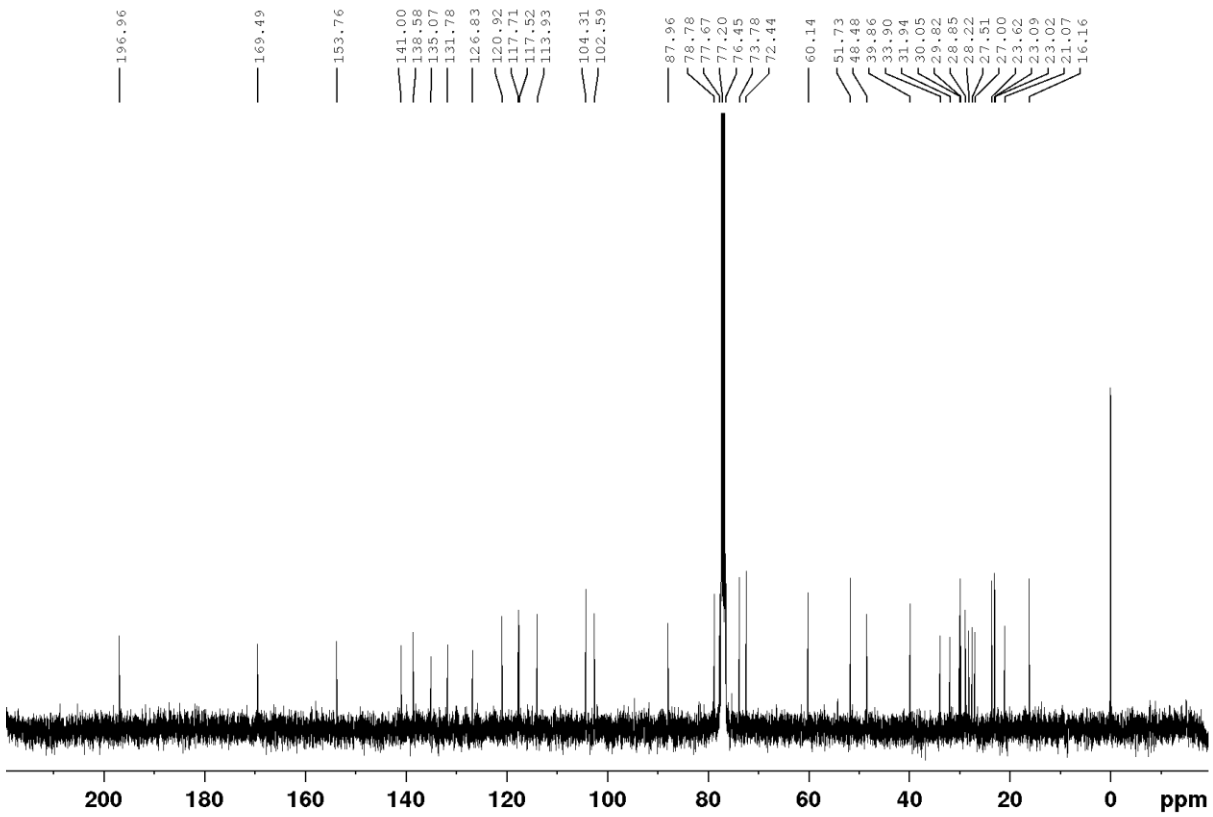

Supplementary Figure 20 | ^{13}C NMR spectrum ($CDCl_3$, 100 MHz) of compound 2

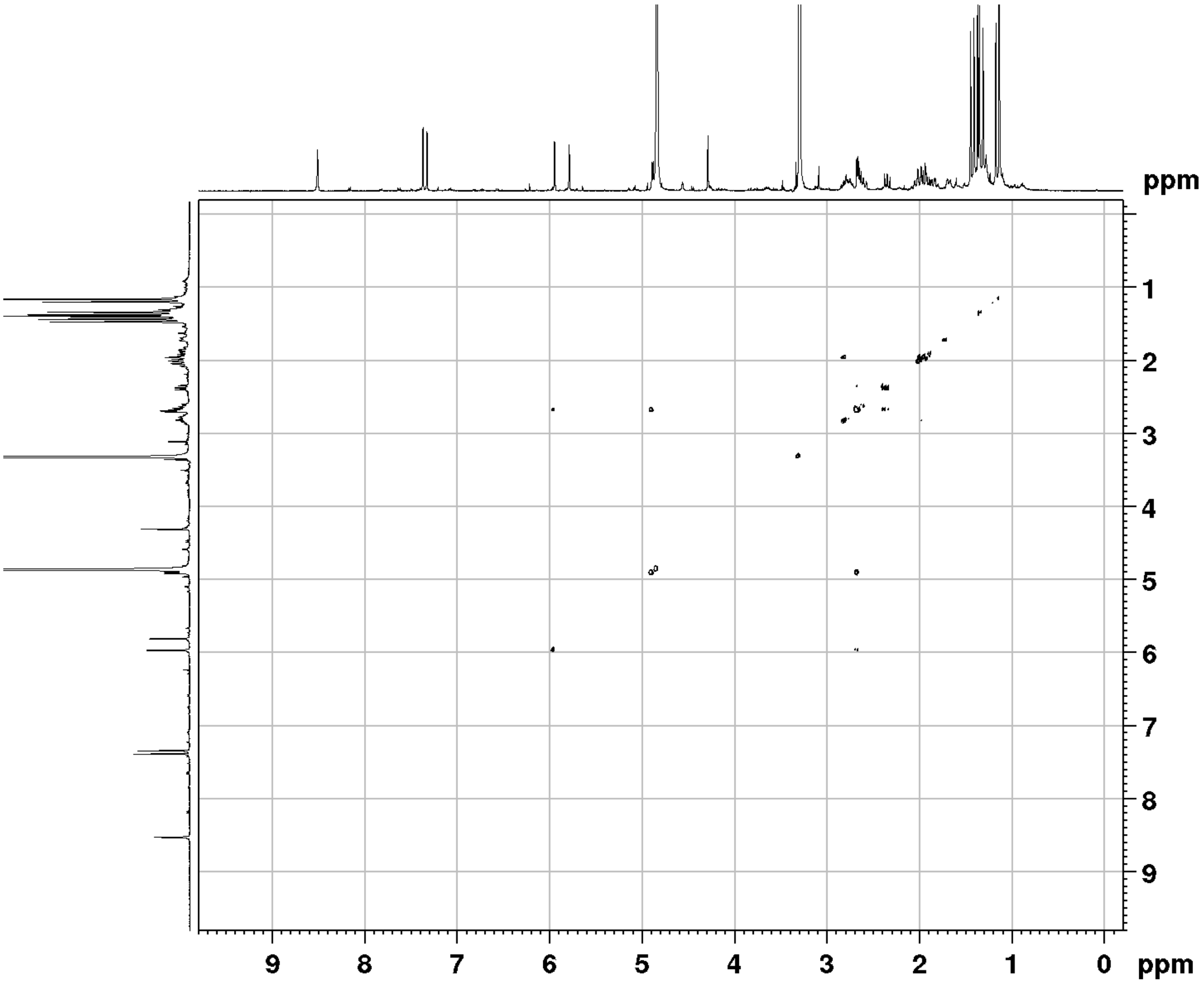

Supplementary Figure 21 | COSY NMR spectrum (MeOD, 400 MHz) of compound 2

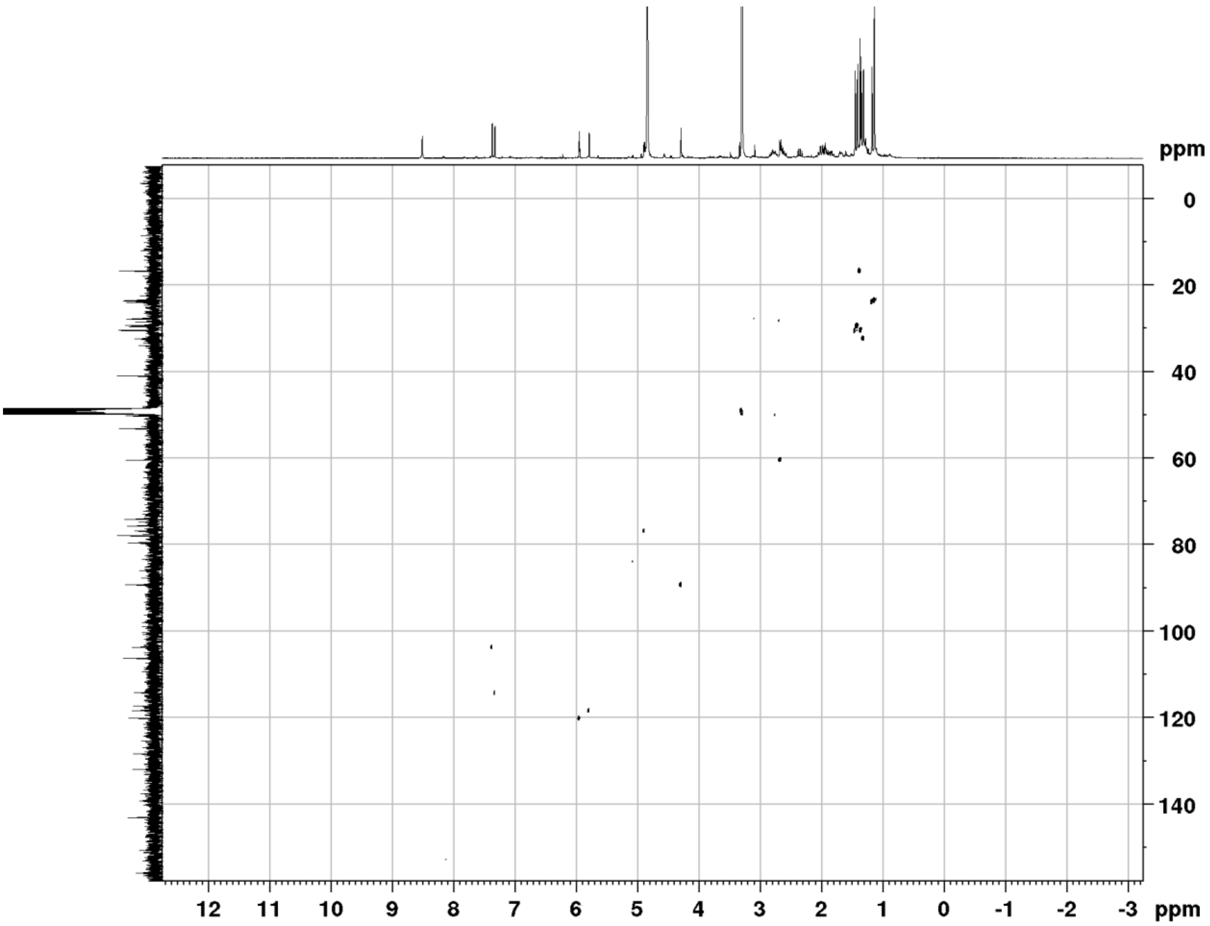

Supplementary Figure 22 | HSQC NMR spectrum (MeOD, 400 MHz) of compound 2

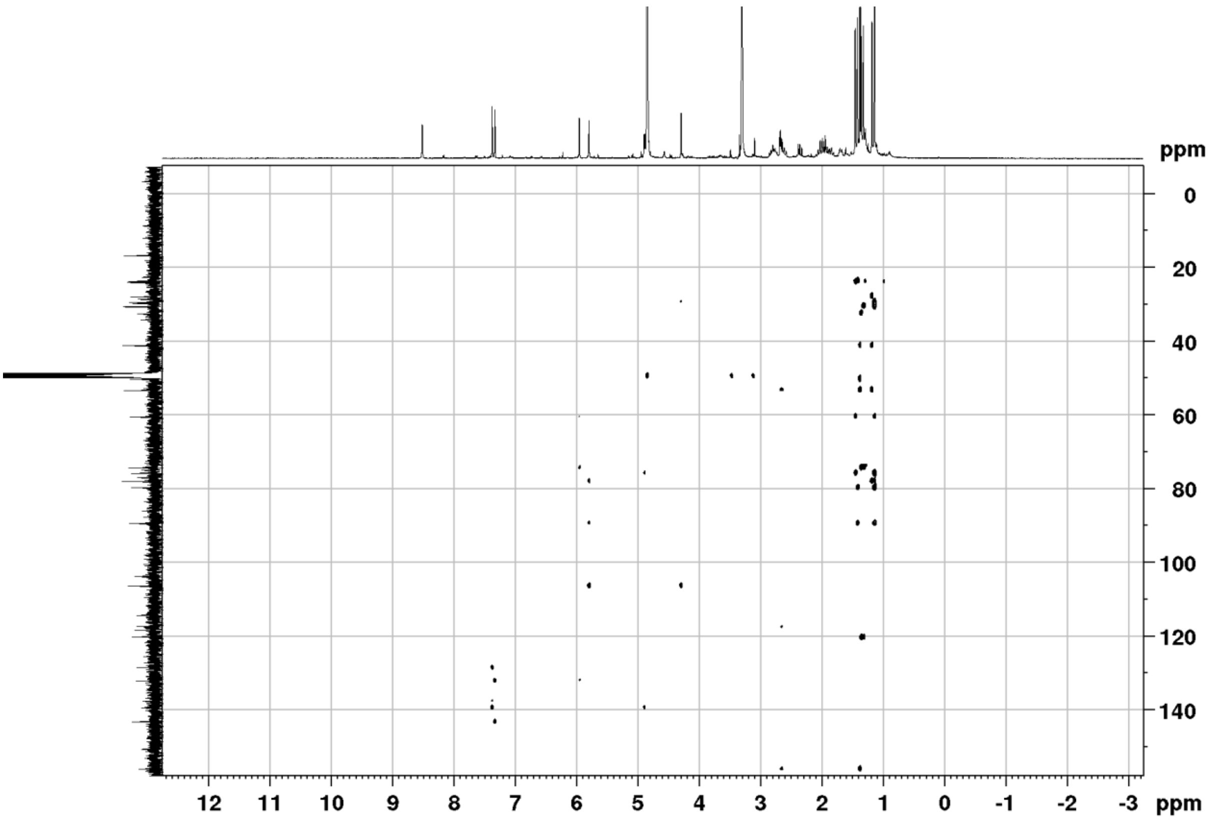

Supplementary Figure 23 | HMBC NMR spectrum (MeOD, 400 MHz) of compound 2

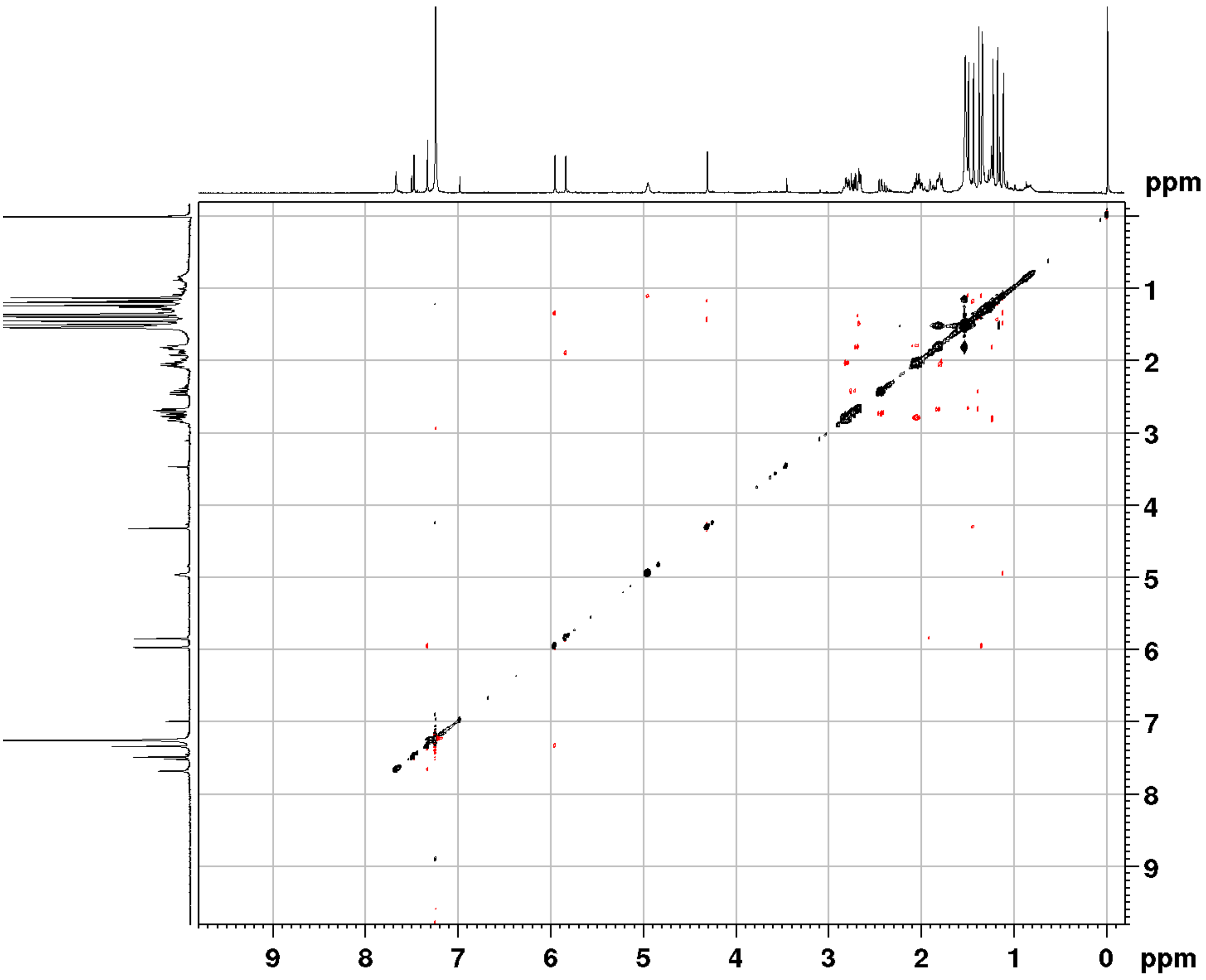

Supplementary Figure 24 | NOESY NMR spectrum ($CDCl_3$, 400 MHz) of compound 2

Supplementary Note 3 | Chemical characterization of melinacidin III (**3**): HR-ESI-MS: *m*/*z* calculated for $C_{30}H_{29}N_6O_7S_4$: 713.0975; observed: 713.0969, $[M+H]^+$: Δ = -0.84 ppm. HRMS data are in agreement with the literature [17].

Supplementary Note 4 | Chemical characterization of chetracin B (**4**): ^{1}H NMR ($CDCl_3$, 400 MHz) δ 7.73 (1H, m) 7.72 (1H, m) 7.20 (1H, m), 7.16 (1H, m), 6.63-6.89 (4H, m), 5.75 (1H, s), 5.66 (1H, s), 5.07 (1H, s), 5.00 (1H, s), 4.10-4.30 (4H, m), 3.23 (3H, s), 3.09 (3H, s); HR-ESI-MS: *m/z* calculated for: $C_{30}H_{29}N_6O_8S_5$: 761.0645; observed: 761.0670 $[M+H]^+$: Δ = 3.28 ppm. Analytical data are in full agreement with literature values [18].

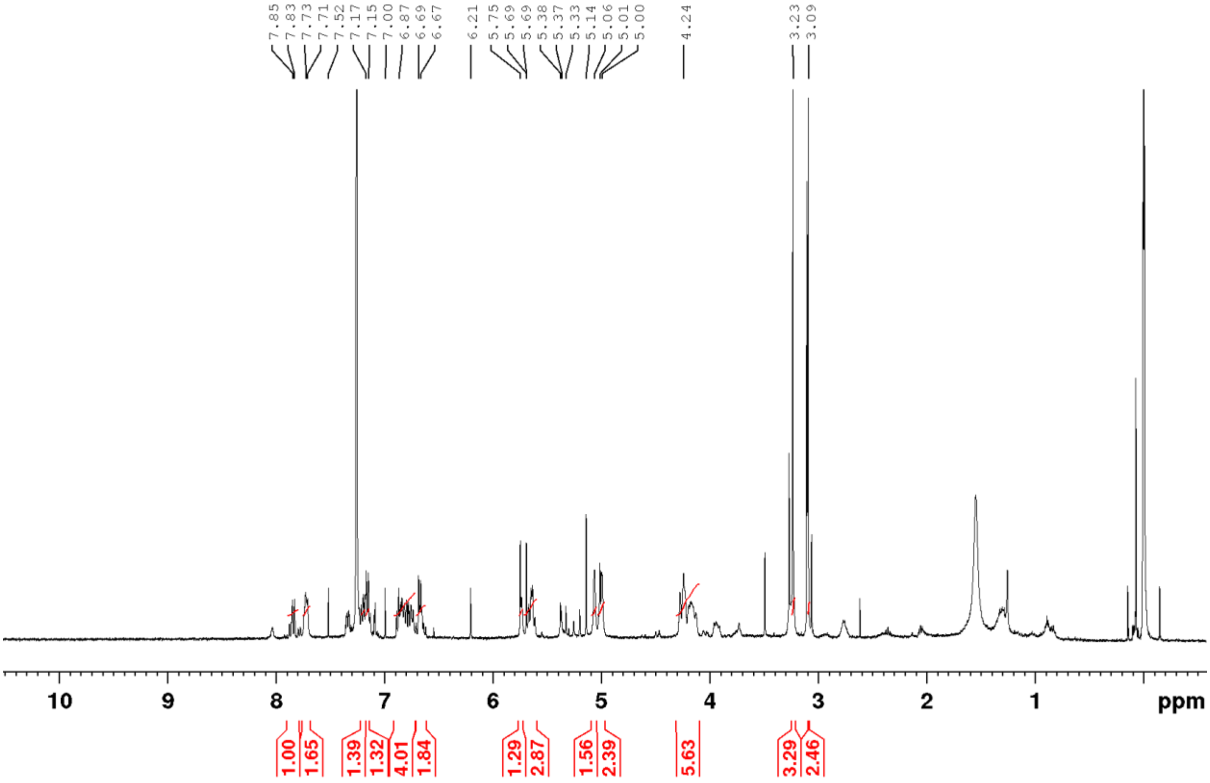

Supplementary Figure 25 | ^{1}H NMR spectrum ($CDCl_3$, 400 MHz) of compound 4

Supplementary Note 5 | Chemical characterization of chetracin C (**5**): ^{1}H NMR (DMSO-d$_6$, 400 MHz) δ 7.49 (4H, d, J = 7.6 Hz), 7.05 (4H, m), 6.51-6.63 (4H, m), 5.38 (2H, d, J = 5.6 Hz), 4.71 (2H, s), 4.01 (2H, m) 3.67 (2H, m), 3.10 (6H, s); HR-ESI-MS: *m/z* calculated for: $C_{30}H_{29}N_6O_8S_6$: 793.0366; observed: 793.0351 $[M+H]^+$: Δ = -1.89 ppm. Analytical data are in full agreement with literature values [18].

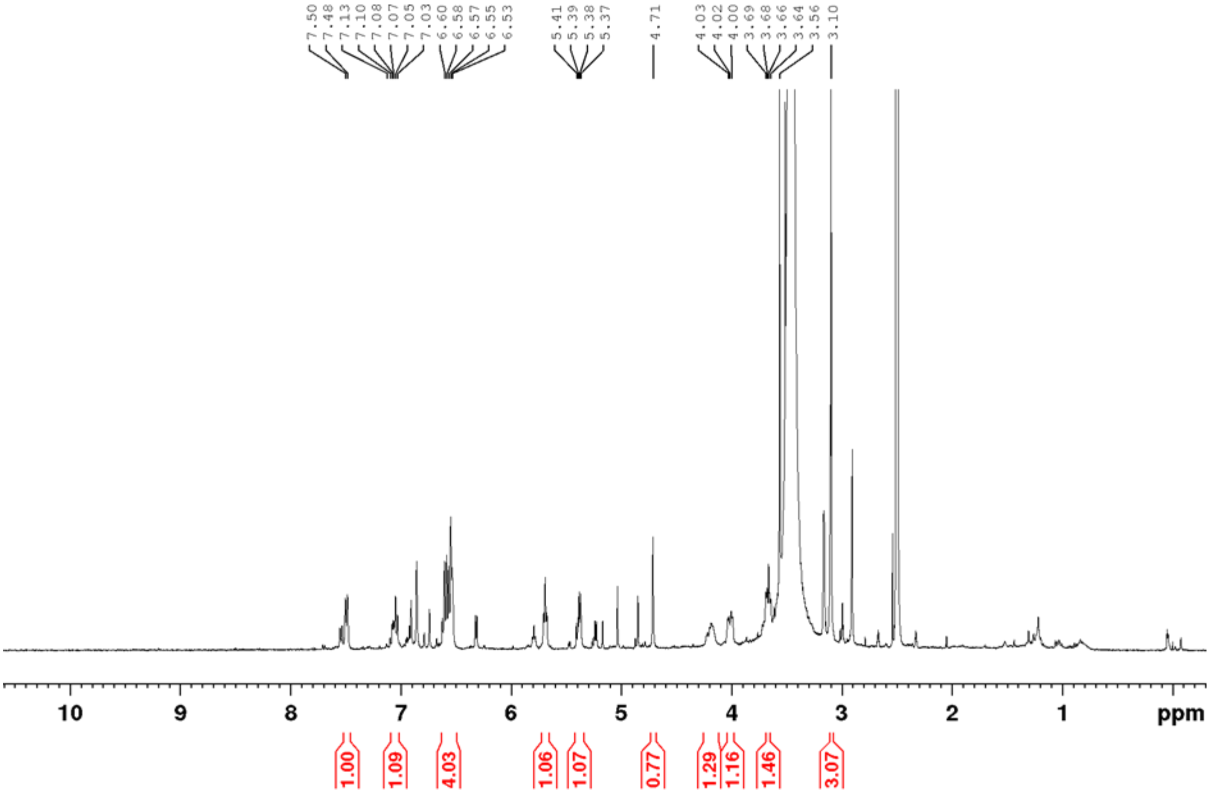

Supplementary Figure 26 | ^{1}H NMR spectrum (DMSO-d$_6$, 400 MHz) of compound 5

Supplementary Note 6 | Chemical characterization of shearinine A (**6**): ^{1}H NMR ($CDCl_3$, 400 MHz) δ 7.57 (1H, s), 7.32 (1H, s), 7.24 (1H, s), 5.91 (1H, d, J = 3.0 Hz), 5.83 (1H, d, J = 0.8 Hz), 4.30 (1H, d, J = 1.1 Hz), 3.47 (1H, OH, s), 3.10 (1H, dd, J_1 =15.3, J_2 = 9.3), 2.91 (1H, m), 2.61-2.83 (5H, m), 2.40 (1H, dd, J_1 =13.1, J_2 =10.7), 1.94-2.07 (4H, m), 1.75-1.81 (2H, m), 1.43 (3H, s), 1.37 (3H, s), 1.35 (3H, s), 1.32 (6H, s), 1.21 (3H, s), 1.17 (3H, s), 1.07 (3H, s); HR-ESI-MS: m/z calculated for: $C_{37}H_{46}NO_5$: 584.3371; observed: 584.3377 $[M+H]^+$: Δ = 1.03 ppm. Analytical data are in full agreement with literature values [19].

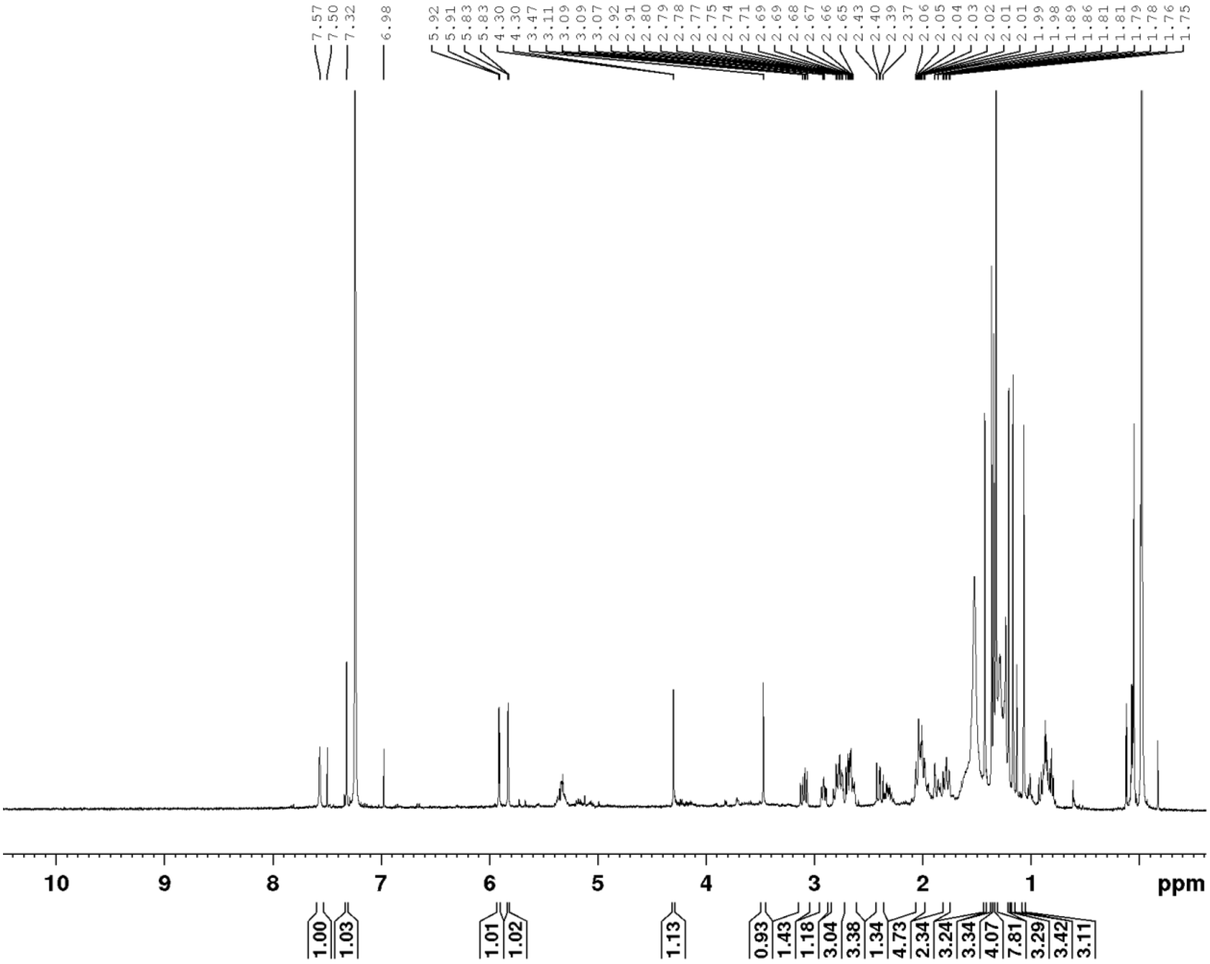

Supplementary Figure 27 | ^{1}H NMR spectrum ($CDCl_3$, 400 MHz) of compound 6

Supplementary Note 7 | Chemical characterization of 22,23-dehydro-shearinine A (**7**): ^{1}H NMR ($CDCl_3$, 400 MHz) δ 7.60 (1H, s), 7.41 (1H, s), 7.19 (1H, s), 6.49 (1H, d, J = 1.8), 6.36 (1H, d, J = 1.8), 5.83 (1H, m), 4.30 (1H, m), 2.82 (2H, m) 2.79 (1H, m), 2.69 (2H, m), 2.64-2.71 (2H, m), 2.04 (1H, m), 2.00 (1H, m), 1.88 (1H, m), 1.78 (1H, m), 1.52 (6H, s), 1.45 (6H, s), 1.43 (3H, s), 1.36 (3H, s), 1.23 (3H, s), 1.17 (3H, s); ^{13}C NMR ($CDCl_3$, 100 MHz) δ 197.0, 169.6, 152.0, 141.9, 138.2, 137.0, 134.0, 129.1, 128.6, 125.4, 121.4, 118.2, 117.8, 110.3, 104.4, 104.2, 87.8, 78.8, 77.5, 73.2, 72.7, 51.6, 48.4, 39.9, 33.9, 31.6, 31.0, 28.9, 28.2, 27.6, 27.0, 23.6, 23.1, 21.1, 16.2 ppm; HR-ESI-MS: m/z calculated for: $C_{37}H_{44}NO_5$: 582.3214; observed: 582.3209 $[M+H]^+$: Δ = -0.86 ppm. All analytical data in agreement with literature values [20].

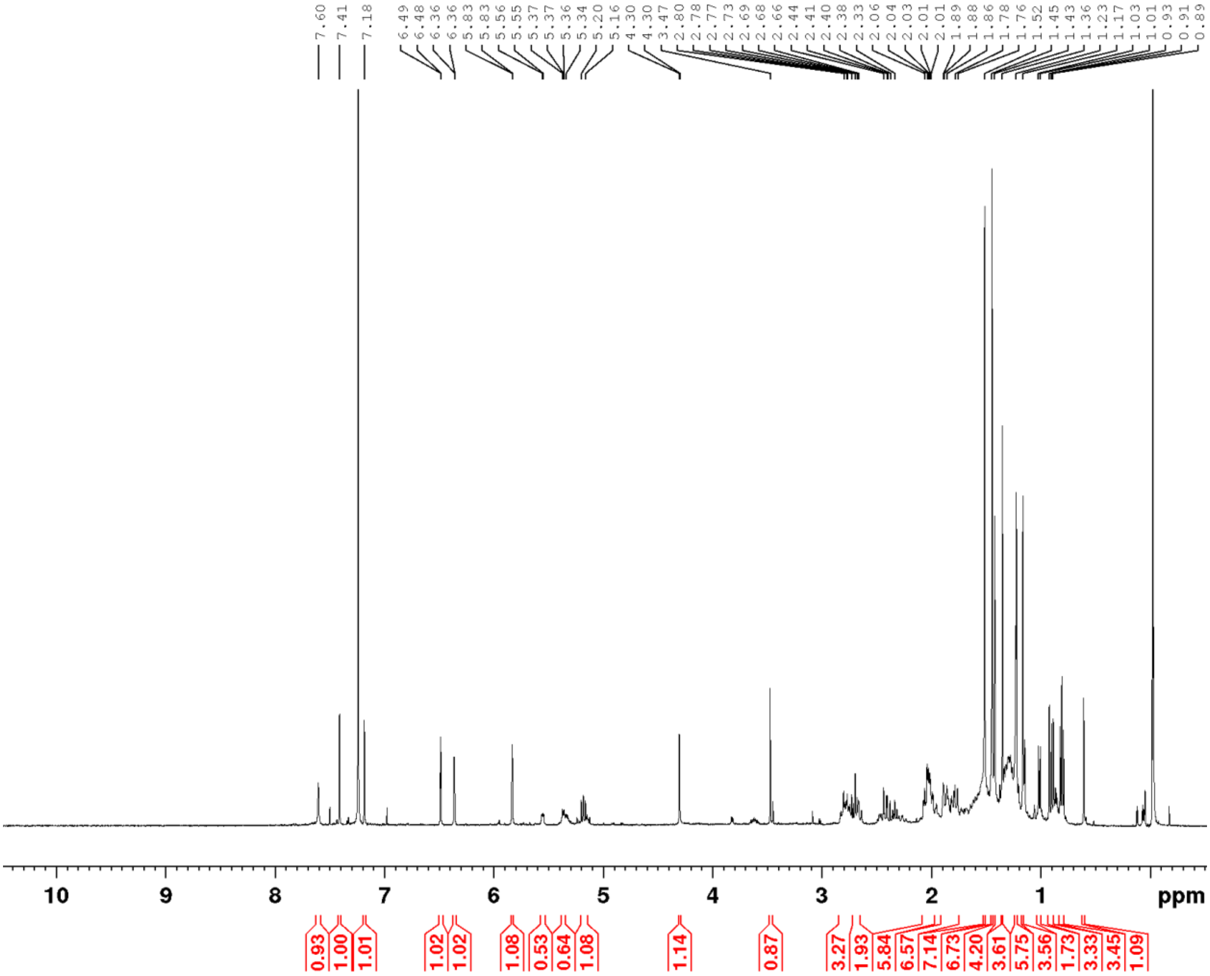

Supplementary Figure 28 | ^{1}H NMR spectrum ($CDCl_3$, 400 MHz) of compound 7

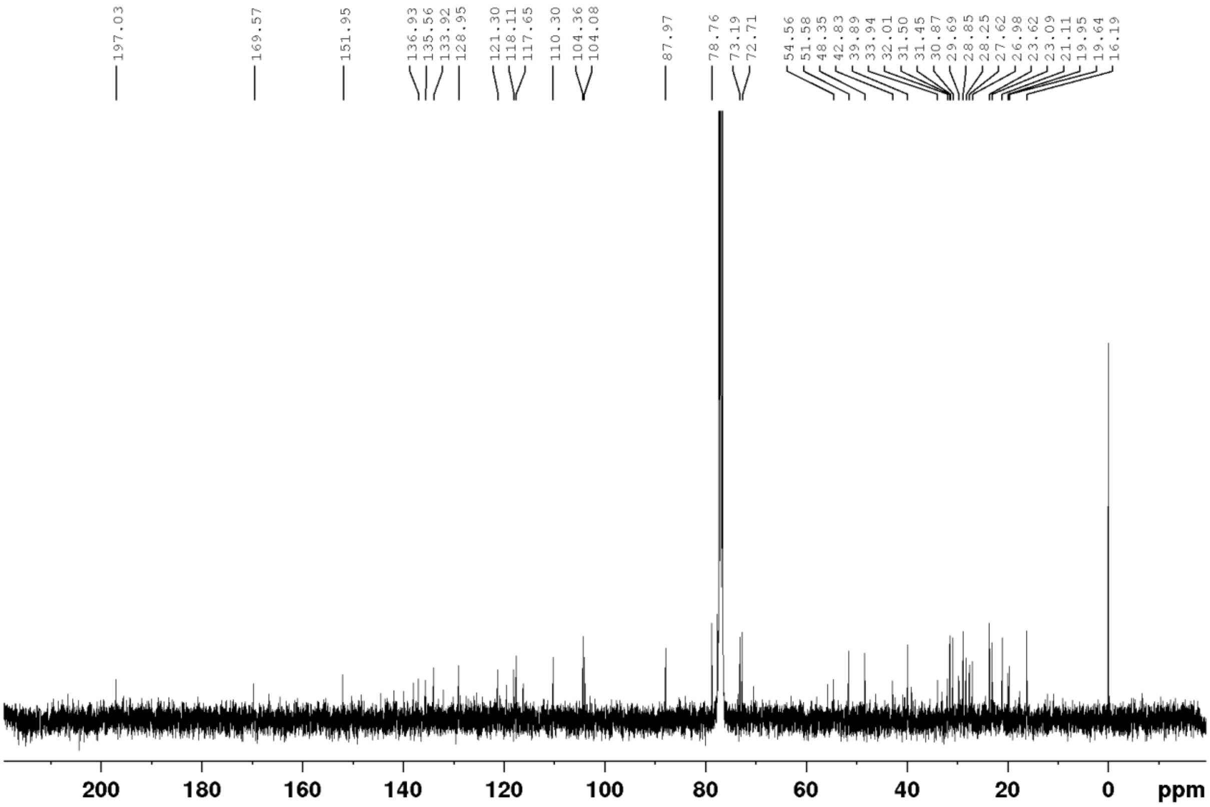

Supplementary Figure 29 | ^{13}C NMR spectrum ($CDCl_3$, 100 MHz) of compound 7

Supplementary Note 8 | Chemical characterization of emodin (**8**). ^{1}H NMR (DMSO-d_6, 400 MHz) δ 12.10 (1H, bs), 12.04 (1H, bs), 7.51 (1H, m), 7.18 (1H, m), 7.12 (1H, d, J = 2.4 Hz), 6.59 (1H, J = 2.4 Hz), 2.41 (3H, s); ^{13}C NMR (DMSO, 100 MHz) δ 189.8, 181.7, 165.8, 164.6, 161.5, 148.4, 135.3, 133.0, 124.3, 120.6, 113.6, 109.1, 109.0, 108.1, 21.7 ppm; HR-ESI-MS: m/z calculated for: $C_{15}H_{11}O_5$: 271.0601; observed: 271.0598 $[M+H]^+$: Δ = -1.11 ppm. Analytical data are in full agreement with literature values [21].

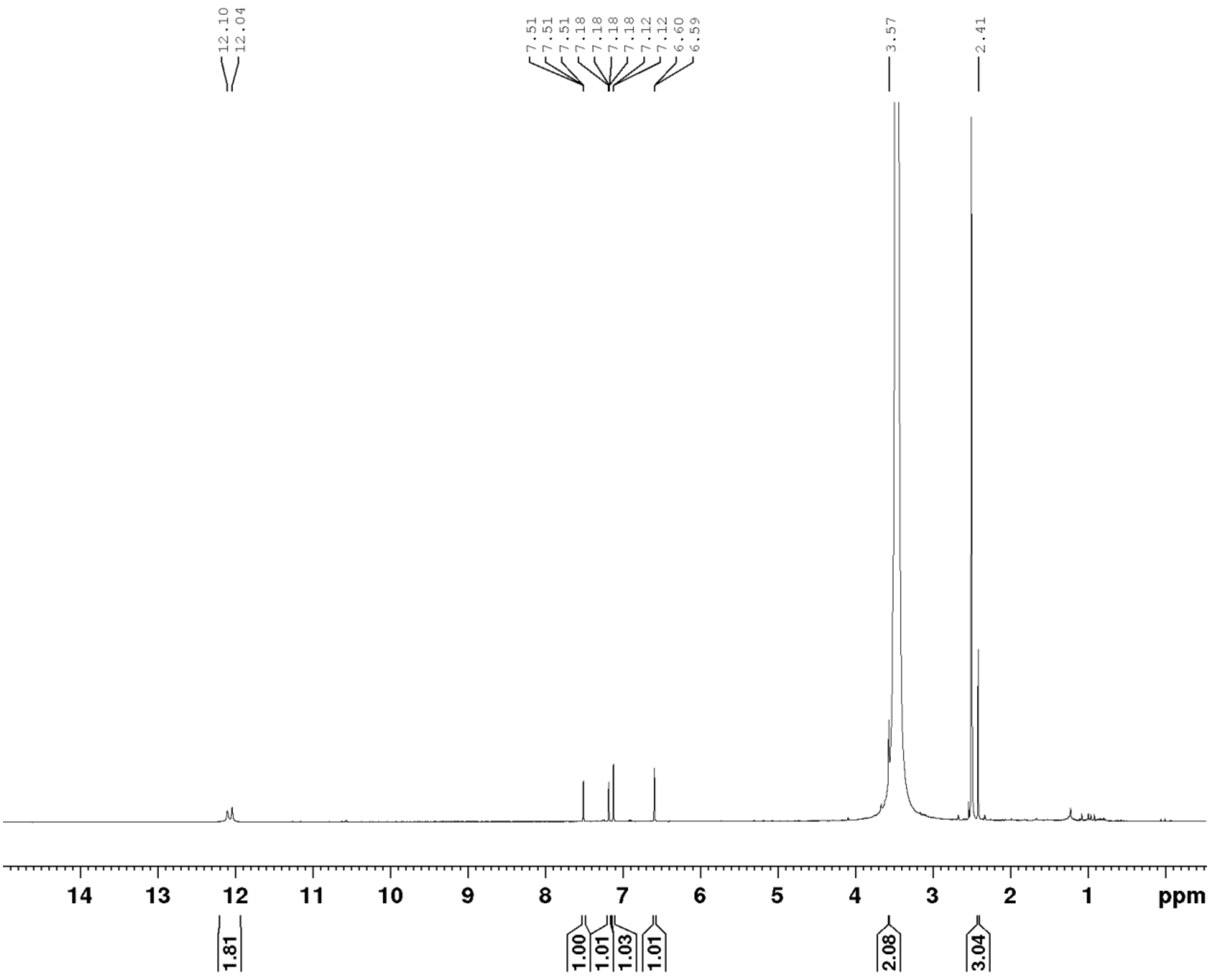

Supplementary Figure 30 | ^{1}H NMR spectrum (DMSO-d_6, 400 MHz) of compound 8

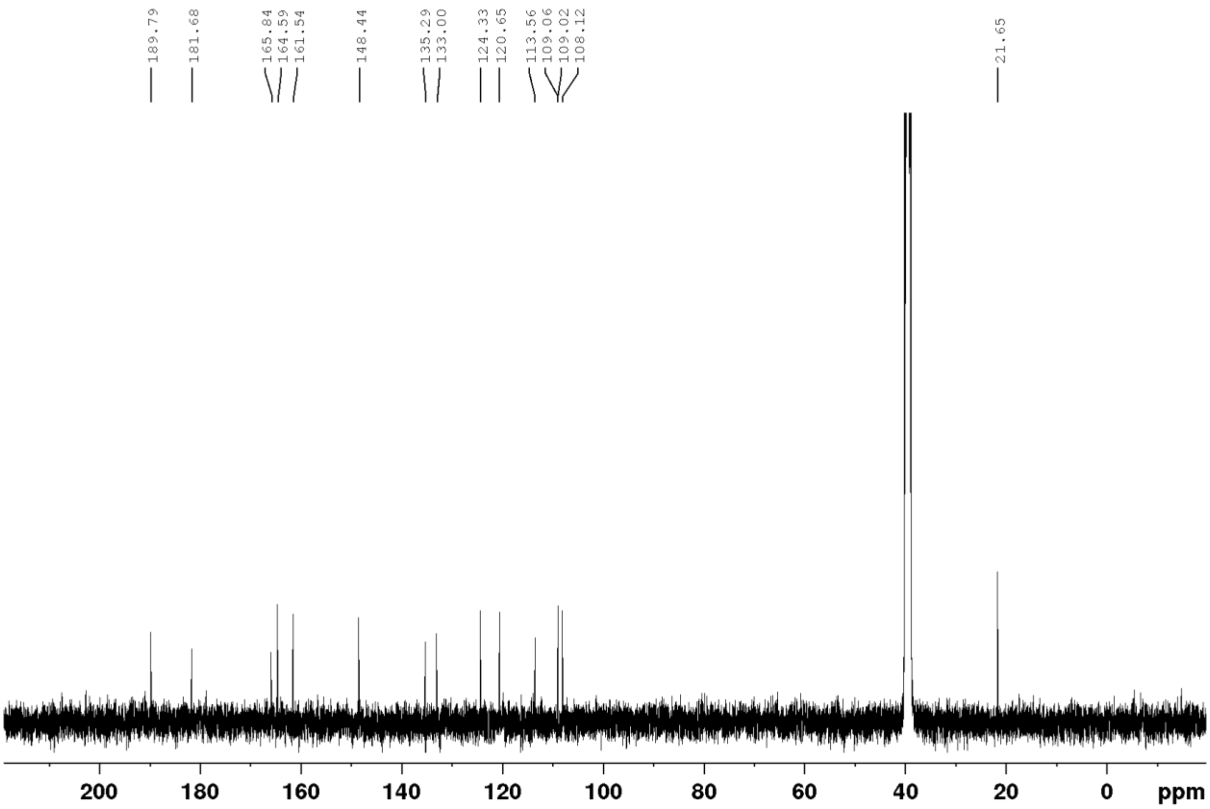

Supplementary Figure 31 | ^{13}C NMR spectrum (DMSO-d_6, 100 MHz) of compound 8

Supplementary References

1. Kieser, T. E. *et al.* Practical *Streptomyces* Genetics. Published by the John Innes Foundation. ISBN 0-7084-0623-8 (2000).
2. Nicholson, M. *et al.* Molecular cloning and functional analysis of gene clusters for the biosynthesis of indole-diterpenes in *Penicillium crustosum* and *P. janthinellum*. *Toxins* **7**, 2701–2722 (2015).
3. Gerken, T. & Walsh, C. T. Cloning and sequencing of the chaetocin biosynthetic gene cluster. *ChemBioChem* **14**, 2256–2258 (2013).
4. Chiang, Y.-M. *et al.* Characterization of the *Aspergillus nidulans* monodictyphenone gene cluster. *Appl. Environ. Microbiol.* **76**, 2067–2074 (2010).
5. Sievers, F. *et al.* Fast, scalable generation of high-quality protein multiple sequence alignments using Clustal Omega. *Mol. Syst. Biol*. **7**, 539 (2011).
6. Chaverri, P., Castlebury, L. A., Overton, B. E. & Samuels, G. J. *Hypocrea/Trichoderma*: species with conidiophore elongations and green conidia. *Mycologia* **95**, 1100–1140 (2003).
7. Meirelles, L. A. *et al.* Shared *Escovopsis* parasites between leaf-cutting and non-leaf-cutting ants in the higher attine fungus-growing ant symbiosis. *R. Soc. Open Sci.* **2**:150257 (2015).
8. Rodrigues, A. *et al.* Microfungal "weeds" in the leafcutter ant symbiosis. *Microb. Ecol.* **56**(4), 604-614 (2008).
9. Kuhls, K. *et al.* Revision of *Trichoderma* sect. *Longibrachiatum* including related teleomorphs based on analysis of ribosomal DNA internal transcribed spacer sequences. *Mycologia*. **89**, 442-460 (1997).
10. Hoyos-Carvajal, L. *et al.* Genetic and metabolic biodiversity of *Trichorderma* from Colombia and adjacent neotropic regions. *Fungal. Genet. Biol.* **46**, 615-631 (2009).
11. Nixon, K. C. WinClada ver. 1.0000. Published by the author, Ithaca, NY, USA (1999-2002).
12. de Man, T. J. B. *et al.* Small genome of the fungus *Escovopsis weberi*, a specialized disease agent of ant agriculture. *Proc. Natl. Acad. Sci. USA* **113**, 3567-3572 (2016).
13. Camacho, C. *et al*. BLAST+: architecture and applications. *BMC Bioinformatics* **10**, 421 (2009).
14. Wang, Y., Tian, R. M., Gao, Z. M., Bougouffa, S. & Qian, P.-Y. Optimal eukaryotic 18S and universal 16S/18S ribosomal RNA primers and their application in a study of symbiosis. *PLoS ONE* **9**:e90053 (2014).
15. Argoudelis, A. D. & Mizsak, S. A. Melinacidins II, III and IV. Structural studies. *J. Antibiot.* **30**, 468–473 (1977).
16. Xu, M. *et al.* Shearinines D–K, new indole triterpenoids from an endophytic *Penicillium* sp. (strain HKI0459) with blocking activity on large-conductance calcium-activated potassium channels. *Tetrahedron* **63**, 435–444 (2007).
17. Watts, K. R. *et al.* Assessing the trypanocidal potential of natural and semi-synthetic diketopiperazines from two deep water marine-derived fungi. *Bioorg. Med. Chem.* **18**, 2566–2574 (2010).
18. Li, L., Li, D., Luan, Y., Gu, Q. & Zhu, T. Cytotoxic metabolites from the antarctic psychrophilic fungus *Oidiodendron truncatum*. *J. Nat. Prod.* **75**, 920–927 (2012).
19. Belofsky, G. N., Gloer, J. B., Wicklow, D. T. & Dowd, P. F. Antiinsectan alkaloids: Shearinines A-C and a new paxilline derivative from the ascostromata of *Eupenicillium shearii*. *Tetrahedron* **51**, 3959–3968 (1995).
20. You, J., Du, L., King, J. B., Hall, B. E. & Cichewicz, R. H. Small-molecule suppressors of *Candida albicans* biofilm formation synergistically enhance the

antifungal activity of amphotericin B against clinical *Candida* isolates. *ACS Chem. Biol.* **8**, 840–848 (2013).

21. Wang, C., Guo, L., Hao, J., Wang, L. & Zhu, W. α-Glucosidase inhibitors from the marine-derived fungus *Aspergillus flavipes* HN4-13. *J. Nat. Prod.* **79**, 2977–2981 (2016).
