# Supplementary material for: Chemical warfare between leafcutter ant symbionts and a co-evolved pathogen
Source: Nat Commun. 2018 Jun 7;9:2208. doi: 10.1038/s41467-018-04520-1 (PMC5992151; doi:10.1038/s41467-018-04520-1)
Supplement: Supplementary file 3 — Description of Additional Supplementary Files [file 41467_2018_4520_MOESM3_ESM.pdf]

Description of Additional Supplementary Files:

Supplementary Movie 1 | A time-lapse video comparing the level of mobility shown by ants fed on different dietary concentrations of compound 2. Concentrations are 0 mM, 0.1 mM, 0.25 mM (bottom left to bottom right Petri dishes) and 0.5 mM, 1 mM and 2 mM (top left to top right Petri dishes). A decrease in ant mobility can be seen from bottom left to top right. *N* = 5 ants per concentration group. The time-lapse vídeo was made over approximately three hours, with 10 second intervals between frames.

Supplementary Movie 2 | A time-lapse video comparing ants fed on either glucose water (left petri dish), or glucose water supplemented with 1 mM of compound 2. *N* = 5 ants per treatment group. The time-lapse vídeo was made over approximately three hours, with 10 second intervals between frames.
